# Co-occurrence of antibiotic residues and Antimicrobial resistance genes in animal manure and agricultural soils from Machakos, Kiambu, and Kajiado Counties, Kenya

**DOI:** 10.1371/journal.pone.0337311

**Published:** 2026-07-24

**Authors:** Richard M. Machoka, Florence Ng’ong’a, Christabel Muhonja, Heshborne Tindih, Zachary Getenga, Sören Thiele-Bruhn

**Affiliations:** 1 Department of Biochemistry, Jomo Kenyatta University of Agriculture and Technology, Nairobi, Kenya; 2 Department of Biological Sciences, School of Pure and Applied Sciences, Machakos University, Machakos, Kenya; 3 Department of Physical Sciences, School of Pure and Applied Sciences, Machakos University, Machakos, Kenya; 4 Department of Soil Sciences, Trier University, Trier, Germany; Fayetteville State University, UNITED STATES OF AMERICA

## Abstract

The excessive and often unregulated use of antibiotics in livestock production and human health has led to the dissemination of antibiotic residues and antibiotic resistance genes (ARGs) posing a serious threat to the environment and public health. This study investigates the co-occurrence and spatial distribution of antibiotic residues and ARGs in livestock manure and agricultural soils from Machakos, Kiambu, and Kajiado counties in Kenya, regions characterized by intensive livestock farming. A total of 180 animal manure and soil samples from 30 farms across the three counties were collected and pooled into 18 samples. Antibiotic residues were extracted and quantified using high-performance liquid chromatography coupled with tandem mass spectrometry (LC-MS/MS). Nine ARGs (*aadA, ermB*, *sul1, tetQ, tetW, dfrA1, blaMOX, blaOXA* and *qnrB* were quantified via absolute and relative qPCR, normalized to *16S rRNA* gene copy numbers. Multivariate analyses, including PCoA and Spearman Correlation, were conducted to explore ARG structure and co-occurrence. Tetracycline, particularly oxytetracycline, was the most abundant antibiotic in manure (up to 1150 ng mL^−1^), especially in pig manure from Kiambu. Sulfadimethoxine was undetectable in nearly all samples except in soil mixed with pig manure from Kajiado county, which showed a concentration above 70 ng mL^−1^, the pig manure from the same county had levels below 2 ng mL^−1^. Sulfadimidine, sulfamethoxanole and erythromycin were not detected in any of the samples. A three-way ANOVA showed that animal source (cattle, pigs, poultry) and sample type (manure, soil) were generally not significant factors for antibiotic concentration. Only oxytetracycline (P = 0.0421, 0.0901) and sulfadimethoxine (P = 0.0904, 0.044, 0.054) showed marginal significance by sample type (P = 0.0785). Antibiotic resistance genes were widely distributed, *with aadA, ermB* and *sul1* being the most prevalent, especially in soils from Kiambu and Machakos. The *tetQ* showed extremely high relative abundance, indicating intense selection pressure from tetracyclines. Strong positive correlations were observed between co-occurring ARGs, including *tetQ* and *tetW* as well as *erm* and *sul1*. The concurrent detection of persistent antibiotic residues and high ARG loads in both manure and soils underscores the urgent need for improved antibiotic stewardship, sustainable manure management, and environmental monitoring in Kenyan agroecosystems.

## Background

Antibiotics are commonly used in the treatment of animal and human diseases; however, excessive usage of antibiotics has led to an increase in antibiotic-resistant microbes and antibiotic-resistance genes (ARGs), which has become a threat to human and animal health [1]. Thus, the World Health Organization (WHO) has listed antimicrobial resistance among the top ten public health threats facing humanity [2]. Globally, about 700,000 deaths result from infections caused by antimicrobial-resistant pathogens annually, with more deaths occurring in Sub-Saharan Africa [3]. This number may rise to about 10 million by 2050 if urgent action is not taken [4,5].

In veterinary medicine, antibiotics make up over 70% of all pharmaceuticals used in livestock production [6], leading to the spread of antimicrobial resistance, especially when used as prophylactic drugs [7]. Furthermore, antibiotics used as ergotropics in low doses to promote animal growth provide perfect growth conditions for antimicrobial-resistant pathogens [8,9]. In this regard, animals may act as a reservoir for antibiotic-resistance genes (ARGs), which then may be transferred to the environment via excrements and consequently to humans through direct handling of animals, direct contact with a contaminated environment and or the food chain [10,9]. The consequences of antibiotic consumption and contamination are exacerbated by environmental pollution, contributing to the environmental resistome. The role and significance of animal waste in antimicrobial resistance (AMR) spread in the environment is therefore crucial and there is need for a One Health approach in dealing with it.

Livestock excrete between 40–90% of the antibiotics either as active metabolites or unchanged isomers or epimers of the parent molecule in feces and urine [11]. Some metabolites end up being more potent, while others such as acetic-conjugated sulphonamides can change back to their parent compounds during manure storage [12].

In cattle production, particularly dairy industry, a range of antibiotics are used including aminoglycosides, chloramphenicol, fluoroquinolones, glycolipids, ionospheres, macrolides quinolones, streptogramins, sulphonamides and tetracyclines. These antibiotics are administered for purposes such as growth promotion, prophylaxis, and in treating diseases like mastitis [13,14]. A study conducted in poultry farms in Thailand reported an average of 303 mg of antibiotics; amoxicillin, colistin doxycycline, oxytetracycline and tilmacoxib administered per bird for routine prophylaxis [14]. A recent study on environmental contamination by antibiotics in China, reported concentrations in surface water ranging from 15.4 to 567.0 ng/l for fluoroquinolones and 292.5 ng/g for tetracycline in sediments and 2.13 × 10^5^ ng/g in soils [15]. A study conducted in Rongai sub county in Kenya, reported 0.06–16.24 mg/kg for oxytetracycline, 13.16–15.23 mg/kg for tetracycline, 0.01–3.97 mg/kg for sulfadiazine and 0.04–4.96 mg/kg for sulfamethoxazole [16].

Antimicrobial resistant genes (ARGs) originating from livestock systems are introduced into the environment primarily through land application of animal’s manure as fertilizer. Previous studied have reported increase in soil ARG abundance in agricultural soils following manure application [17]. Comparative analysis between manured and non-manured soils have demonstrated ARG abundances as high as 28,000-fold greater in amended soils, with relative abundance ranging from 10^-4^ to 10^-1^ copies per 16S rRNA gene, corresponding to to absolute abundances of approximately 10⁶–10^10^ copies per gram of soil. Notably, even single manure application event can result in the persistent of elevated ARG levels for upto 120 days [18]. In addition to cellular DNA, ARGs have been detected in extracellular DNA (eDNA) and bacteriophages present in animal manure, indicating that horizontal gene transfer via transformation and transduction plays a significant role in ARG dissemination. Furthermore, several antibiotics commonly used in animal husbandry are known to enhance horizontal gene transfer by inducing DNA damage and activating the bacterial SOS response, thereby facilitating the spread and maintenance of antimicrobial resistance in environmental microbial communities [19,20].

In recognition of the importance of AMR to public health, both human and environmental, and in alignment with the Global Action Plan on AMR, the Kenyan government released a National Action Plan on AMR in 2017. This plan was revised in 2023 to formulate a comprehensive response to AMR by involving all relevant stakeholders [21]. In this report, it is recognized that combating AMR requires a One Health approach from all sectors to address the gaps in the fight against AMR, which include a lack of knowledge, poor surveillance, and misuse of antibiotics by farmers [22]. About 90% of all antibiotic purchases in Kenya for veterinary purposes are used for prophylactic rather than curative purposes [23]. The Kenya Veterinary Board estimated that over 33% of antibiotics available for animal use are substandard or counterfeit, further complicating the situation [24]. Antibiotic residues have been found in various animal products sold in markets in Kenya, including chicken meat [25] and milk sold by vendors in Kibera, where beta-lactams and oxytetracyclines were found in over 66% of unpasteurized milk [26]. Sulfonamide residues have been isolated from the dairy value chain in Nakuru, Kenya [27].

Consequently, it is urgently needed to elucidate the concentrations of various antibiotic residues and the ARG profiles from different animal manure and representative agricultural soils in Kenya. This study therefore aims at analysing the antibiotic residues and the antimicrobial resistance gene profiles in the three highly agricultural counties in Kenya where antibiotics are routinely used for various livestock species.

## Materials and methods

### Study site

This study was conducted in three counties in Kenya: Machakos, Kiambu and Kajiado. Machakos borders Nairobi and Kiambu Counties to the west, Kitui County to the East, Makueni county to the South, Embu County to the North Kajiado to the southwest. It is located between latitudes 0^o^ 45’ and 1^o^31’ South and between longitudes 36° 45’ and 37° 45’ East. Administratively, Machakos is divided into about 40 wards and it covers about 6,208 km^2^ with an estimated population of 1.42 million. Subsistence farming is the main economic activity in the county where mixed farming is practiced [28].

Kiambu County is located in the central region and covers a total area of 2,543.5 km^2^ with 476.3 km^2^ under forest cover according to the 2009 Kenya Population and Housing Census. Kiambu County borders Nairobi and Kajiado Counties to the South, Machakos to the East, Murang’a to the North and northeast, Nyandarua to the northwest, and Nakuru to the West. The county lies between latitudes 00 25’and 10 20’South and between longitudes 360 31‘and 370 15‘East. It has a population of about 1.76 million people. Small-scale farming is the main economic activity and zero grazing is practiced given the limited farm sizes in the county. Animals reared include cattle, sheep and goats, poultry and pigs. Growth in livestock farming was motivated by the ready market in the urban centres and the availability of local food processing factories [29].

Kajiado County covers an area of 21,900.9 square kilometers (km^2^). It is located in the southern part of Kenya and borders Nairobi County to the North East, Narok County to the West, Nakuru and Kiambu Counties to the North, Taita Taveta County to the South East, Machakos and Makueni Counties to the North East and East respectively, and the Republic of Tanzania to the South. It is situated between longitudes 360 5’ and 370 5’ East and between latitudes 10 0’ and 30 0’ South. The main economic activities carried out mainly in Kajiado are pastoralism, wholesale and retail trade, and mining, especially soda ash in Magadi and marble in Kajiado central. Agriculture includes both horticulture and small-scale farming [30].

A study conducted to analyse the soil chemical properties showed a significant variation among Kiambu, Machakos and Kajiado counties which reflects the differences in agroecological and land use practices. Kiambu exhibited more acidic soils (pH 5.8) and higher levels of total nitrogen, organic carbon, Fe, and Zn, consistent with its predominantly cultivated croplands and long-term fertilizer application. In contrast, Kajiado and Machakos—both semi-arid to arid counties with historically limited crop production—had more alkaline soils (pH 7.0–7.7) and generally higher concentrations of Ca, Mg, CEC, K, Na, and B [31]. These differences align with the distinct agricultural histories of the counties: Kiambu’s intensive crop cultivation has enhanced nutrient accumulation, while Kajiado and Machakos are transitioning from predominantly pastoral systems to mixed farming in response to increasing population pressure and expanding food production in semi-arid regions [32,33].

### Sample size determination, sampling design, and sample collection

This study employed a stratified random sampling technique, with the counties serving as strata. In each stratum (Machakos, Kiambu and Kajiado) purposive sampling was used to select ten animal farms of interest, which were chosen for their high or frequent use of antibiotics. In the end, random sampling was done, with samples collected at the farm owners’ discretion.

According to anecdotal data collected from the three-county agriculture field extension officers, there are approximately between 100 and 150 farms in each of the three counties that rear cattle, pigs and poultry either together in one farm or separately, making the total population about 330 farms. The sample size was calculated using the following equation [34].


$$\begin{array}{c} n=\;\frac{N}{\text{1}\;+\text{N}{\left(\text{e}\right)}^{\text{2}}} \end{array}$$

Where:

n = sample size

N is the population size 330

e= level of precision=0.05

therefore


$$\begin{array}{c} \text{n}=\text{330}/\text{1}\;+\text{330}{\left(\text{0}.\text{05}\right)}^{\text{2}}\;=\text{ 180} \end{array}$$

In accordance with the calculated sample size, a total of 180 samples were collected. To this end, 10 manure samples were taken from each of the three livestock groups (cattle, pigs and poultry) in each county, along with soil from 30 sites that were exposed to the manure. This gave a total of 60 samples per county. For the subsequent analysis, the 180 samples were grouped into 18 clusters, i.e., manure from the same county and livestock group as well as soil from the same county and receiving manure from the same livestock group. The samples from the same cluster were pooled into a single composite sample. The purpose of pooling was to even out potential extreme values in individual samples and thereby make the relation between manure and soil contamination more reliable, which can be distorted by the temporally and spatially disparate environmental substrates that were sampled. However, pooling also reduced the significance of results.

### Sample collection

Sterile spatulas and zip-lock bags were used to collect animal fresh manure and the corresponding agricultural soil samples associated with each livestock group. Soil was sampled at a depth of 5–10 cm below the surface. To minimize cross-contamination, fresh sterile gloves were worn for each sampling event. All samples were placed in a cooler box at 4^o^C and transported to the Biochemistry laboratory at Jomo Kenyatta University of Agriculture and Technology where they were stored at −20°C util further processing.

### Extraction and quantification of antibiotic residues in soil and manure samples

Extraction experiments were performed by weighing 1 g of each manure and soil sample into a sterile 50 mL polypropylene centrifuge tube, followed by the addition of 10 mL of extraction solvent which consisted of 2,50 mL 0.1M EDTA-McIlvaine buffer (pH 4), 3.75 mL Acetonitrile + 0.1% formic acid and 3.75 mL MeOH + 0.1% formic acid. Each tube was vortexed vigorously for one minute, followed by ultrasonic extraction using a sonotrode for 15 minutes. After sonication, the samples were centrifuged using at 3,000 × g for 5 minutes (Universal 320 Hettich centrifuge, Germany). A volume of 10 mL of the clear supernatant was carefully transferred into a 20 mL amber glass vial. The supernatant was then evaporated under a gentle stream of nitrogen gas at 40°C until the volume was reduced to approximately 5 mL. To further improve analyte retention during solid-phase extraction (SPE), the supernatant was diluted with 13 mL of 0.1M EDTA solution.

Oasis HLB SPE cartridges were preconditioned sequentially with 5 mL of methanol and 5 mL of EDTA-McIlvaine buffer. The cartridges were loaded with the sample, then washed with 5 mL of bi-distilled water to remove residual impurities and dried under a mild vacuum for 1 minute. Elution was performed in two steps using 2 × 3 mL of methanol, and the eluates were evaporated to dryness under nitrogen gas at 40 °C, then reconstituted in 1 mL methanol. The reconstituted samples were transferred into 2 mL Eppendorf tubes and centrifuged at 12,000 × g for 30 minutes. The clarified extracts were transferred into 1.5 mL amber vials and stored at −20°C until further analysis.

Target analysis of the antibiotics was focused on a set of nine selected antibiotics, i.e., amoxicillin, enrofloxacin, erythromycin, lincomycin, tylosin, sulfadimethoxine, sulfamethoxazole, oxytetracycline, and tetracycline. Antibiotics were analysed as was reported by Ngigi et al. (2018) [35] using an LC-ESI-triple MS (API 3200, Applied Biosystems/MDS Sciex Instruments, Toronto, Canada) coupled to a Shimadzu LC-20 HPLC (Shimadzu, Duisburg, Germany). The stationary phase was a Sunfire C18, 3.5 μm, 3.0 × 20 mm chromatographic column. The eluent consisted of 0.1 M HCOOH in water (solvent A) and 0.1 M HCOOH in methanol (solvent B) and was delivered at 1 mL min^−1^ in a gradient program. The analytical data were assessed using the software Analyst 1.4.2 (Applied Biosystems/MDS Sciex Instruments). Compound quantification was done by summarizing the signals of the different mass transitions. The ratio of two mass transition signals was used for compound identification. Sulfapyridine and chloramphenicol or isotopically labelled antibiotic standards were used as internal standards and external standards of each antibiotic were used for calibration. The limit of detection of the analytical method was 5 μg L^−1^ and the limit of quantification (LOQ) was 10 μg L^−1^.

### DNA extraction

DNA was extracted using the Quick-DNA™ Fecal/Soil Microbe Miniprep Kit from Zymo Research Inc. (Germany) following the manufactures instructions. Briefly, 150 mg of manure and 250 mg of soil sample was added to a ZR BashingBead™ Lysis Tube (0.1 & 0.5 mm). Subsequently, 750 µl of BashingBead™ Buffer was introduced into the tube. The tube was secured in a vortex mixer and vortexed for 40 minutes to lyse all the cells. Followed by centrifugation at ≥ 10,000 × g for 1 minute. 400 µl of the supernatant was transferred to a Zymo-Spin™ III-F Filter in a Collection Tube and centrifuged at 8,000 × g for 1 minute. Then, 1,200 µl of Genomic Lysis Buffer was added to the filtrate in the Collection Tube and mixed well. A total of 800 µl of this mixture was transferred to a Zymo-Spin™ IICR Column in a Collection Tube and centrifuged at 10,000 x g for 1 minute. The flow-through was discarded, and the step was repeated to ensure maximum binding. Next, 200 µl of DNA Pre-Wash Buffer was added to the Zymo-Spin™ IICR Column, which was placed in a new Collection Tube, and centrifuged at 10,000 × g for 1 minute. Following this, 500 µl of g-DNA Wash Buffer was added to the Zymo-Spin™ IICR Column and centrifuged at 10,000 × g for 1 minute. The Zymo-Spin™ IICR Column was then transferred to a clean 1.5 ml microcentrifuge tube, and 100 µl of DNA Elution Buffer was directly applied to the column matrix. The tube was centrifuged at 10,000 × g for 30 seconds to elute the DNA. A Zymo-Spin™ III-HRC Filter was placed in a clean Collection Tube, and 600 µl of Prep Solution was added. This mixture was centrifuged at 8,000 × g for 3 minutes. Finally, the eluted DNA was transferred to a prepared Zymo-Spin™ III-HRC Filter in a clean 1.5 ml microcentrifuge tube and centrifuged at 16,000 × g for 3 minutes to complete the purification process.

### Quantitative PCR for resistance genes

Absolute quantification of selected antibiotic resistance genes (ARGs)—*blaMOX, blaOXA, qnrB, aadA, dfrA1, sul1, tetQ, tetW,* and *ermB*—was performed using the qTOWER³ Auto real-time PCR system (Analytik Jena, Germany). Gene-specific primers targeting conserved regions of each ARG were designed (S1 File) based on validated reference sequences from NCBI GenBank to ensure specificity and amplification efficiency. Primers were synthesized by General Biosystems Inc. (USA) using high-fidelity phosphoramidite chemistry and purified by high-performance liquid chromatography (HPLC). For absolute quantification, synthetic double-stranded DNA standards encompassing the full target amplicon regions were generated by GeneUniversal Inc. (USA). These standards were accurately quantified via spectrophotometry and serially diluted to construct standard curves for each gene. qPCR amplification reactions were carried out in 20 µL volumes using the innuMIX qPCR DSGreen Standard master mix (Analytik Jena, Germany), a dye-based mix optimized for high sensitivity and specificity. Each reaction consisted of 10 µL of innuMIX qPCR DSGreen master mix, 1 µL of template DNA, 0.8 µL of forward primer, 0.8 µL of reverse primer, and 7.6 µL of molecular-grade nuclease-free water. The PCR thermal cycling conditions comprised an initial denaturation at 95°C for 10 minutes, then 40 cycles of denaturation at 95°C for 20 seconds, annealing at 60°C for 1 minute and extension at 72°C for 30 seconds, with product melting between 60°C to 95°C for 15 minutes. The goodness of the qPCR analyses was validated by the quality of the amplification curves, the melting temperatures, and the regression of the calibration curves (R² > 0.98).

### Data analysis

The average concentration of the antibiotic residues was calculated and visualized using bar charts to provide a summary of their distribution across samples. A three-way ANOVA was employed to analyse the variation in antibiotic concentrations across three categorical factors: sample type (manure and soil), livestock group (cow, pig, and chicken), and county of origin (Machakos, Kiambu, and Kajiado). It was previously verified that the data are normally distributed. Homogeneity of variance was determined using Levene’s test. The level of full statistical significance was set as usual at α = 0.05. In addition, a level of marginal significance was defined with α ≤ 0.1, as is common in exploratory studies with a limited sample size (e.g., [36]

Absolute quantification of antimicrobial resistance genes (ARG) was performed and the values were normalized to 16S rRNA gene copies to obtain relative quantification. The normalization accounted for variation in microbial biomass and the total bacterial load which in turn allowed for an accurate cross sample comparison. This normalized data was subsequently used to evaluate the composition structure of the resistome, to estimate the ARG diversity and richness and to explore the relationship between ARGs and environmental factors.

Principal Coordinate Analysis (PCoA) was conducted to explore patterns in ARG composition, while spearman correlation analysis was employed to assess the co-occurrence and potential relationship among different ARGs. Multivariate analyses, i.e., non-metric multidimensional scaling (NMDS) and principal component analysis (PCA) were conducted to assess clustering by sample type or county. All statistical analysis and data visualization were carried out in R version 4.5.1.

### Ethical statement

Ethical approval and research permit was sought and granted from The National Commission for Science, Technology and Innovation (NACOSTI) in Kenya under License No: NACOSTI/P/23/29612.

## Results

### Occurrence of antibiotic residues in manure and soil

**Fig 1:**
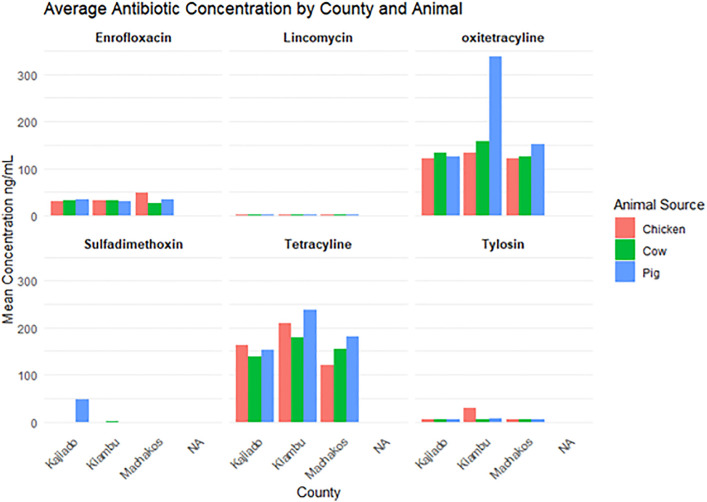
Average antibiotic concentration in liquid manure by counties (Kajiado, Kiambu and Machakos) and livestock group (cattle, pigs and poultry).

From the nine target analytes, seven antibiotics were detected in the different samples (Table 1). The total concentration of antibiotics in the samples pooled by county showed distinct spatial patterns in residue accumulation across Machakos, Kiambu, and Kajiado. Overall, the highest total concentration of antibiotics was measured in the samples from Kiambu, followed by Machakos and Kajiado showing comparatively lower levels (S2 File) (Fig 1).

**Table 1:** Summary of three-way ANOVA results evaluating the effects of animal source, sample type and county on the residual concentrations of seven veterinary antibiotics in manure and soil samples.

Source of Variation	Antibiotic	Df	Sum Sq	Mean Sq	F value	Pr(>F)
**livestock group**	Amoxicillin	2	143	71.4	0.239	0.788
	Enrofloxacin	2	143	71.4	0.239	0.788
	Lincomycin	2	0.42	0.212	0.086	0.917
	Tylosin	2	149	74.75	0.836	0.439
	Sulfadimethoxine	2	3990	1995	2.513	0.090
	Oxytetracycline	2	78,984	39,492	2.401	0.100
	Tetracycline	2	17,280	8,640	0.853	0.432
**sample type**	Amoxicillin	1	488	488.3	1.635	0.206
	Enrofloxacin	1	488	488.3	1.635	0.206
	Lincomycin	1	0.05	0.0512	0.021	0.886
	Tylosin	1	169	169	1.889	0.175
	Sulfadimethoxine	1	1875	1875.2	2.362	0.130
	Oxytetracycline	1	26,508	26,508	1.611	0.210
	Tetracycline	1	32,585	32,585	3.216	0.079
**county**	Amoxicillin	2	17	8.5	0.029	0.972
	Enrofloxacin	2	17	8.5	0.029	0.972
	Lincomycin	2	0.24	0.1218	0.050	0.952
	Tylosin	2	308	154.03	1.722	0.188
	Sulfadimethoxine	2	3990	1995	2.513	0.090
	Oxytetracycline	2	110,599	55,300	3.362	0.042
	Tetracycline	2	42,651	21,326	2.105	0.132
**livestock group × sample type**	Amoxicillin	2	51	25.7	0.086	0.918
	Enrofloxacin	2	51	25.7	0.086	0.918
	Lincomycin	2	0.02	0.0102	0.004	0.996
	Tylosin	2	317	158.57	1.773	0.180
	Sulfadimethoxine	2	4123	2061.6	2.597	0.084
	Oxytetracycline	2	82,835	41,417	2.518	0.090
	Tetracycline	2	9,713	4,857	0.479	0.622
**livestock group × county**	Amoxicillin	4	993	248.1	0.831	0.511
	Enrofloxacin	4	993	248.1	0.831	0.511
	Lincomycin	4	0.13	0.033	0.013	1.000
	Tylosin	4	363	90.76	1.015	0.408
	Sulfadimethoxine	4	8358	2089.6	2.632	0.044
	Oxytetracycline	4	128,912	32,228	1.959	0.114
	Tetracycline	4	14,448	3,612	0.357	0.838
**sample type × county**	Amoxicillin	2	675	337.7	1.131	0.330
	Enrofloxacin	2	675	337.7	1.131	0.330
	Lincomycin	2	0.07	0.036	0.015	0.986
	Tylosin	2	333	166.7	1.864	0.165
	Sulfadimethoxine	2	4123	2061.6	2.597	0.084
	Oxytetracycline	2	23,719	11,859	0.721	0.491
	Tetracycline	2	9,669	4,835	0.477	0.623
**livestock group × sample type × county**	Amoxicillin	4	320	79.9	0.268	0.898
	Enrofloxacin	4	320	79.9	0.268	0.898
	Lincomycin	4	0.77?	0.192	0.078	0.989
	Tylosin	4	644	161.05	1.801	0.142
	Sulfadimethoxine	4	7874	1968.4	2.48	0.055
	Oxytetracycline	4	171,891	42,973	2.612	0.045
	Tetracycline	4	19,959	4,990	0.492	0.741

**Fig 2:**
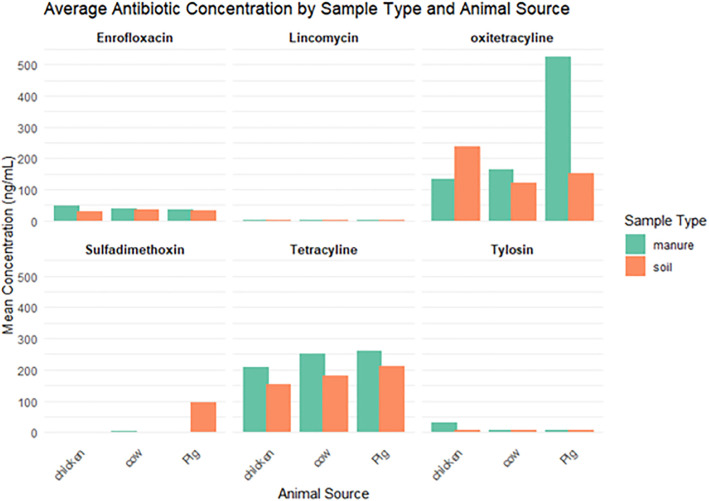
Average concentration of antibiotics by sample type (manure and soil) and manure source (cow, pig and chicken).

Enrofloxacin was detected in all three counties, in both soil and manure samples originating from the excreta of the three groups of livestock animals. Thereby, concentrations of antibiotics were higher in manure than in soils. Chicken manure from Machakos exhibited the highest median concentration (~65 ng mL^−1^) and soil laced with cow manure from Machakos was below detection limits. Lincomycin was detected in low quantities with chicken manure from Kajiado having the highest concentrations of 3.53 ng mL^−1^. Oxytetracycline concentrations were high in all the tested samples across the three counties. The highest average concentration, at over 665 ng mL^−1^, was found in pig manure from Kiambu County. Tetracycline was also determined in consistently high concentration in all samples, with pig manure from Kiambu County showing the highest concentration of more than 200 ng mL^−1^. As shown in Fig 2, sulfadimethoxine was below the limit of detection in most samples, except pig manure from Kajiado County, where the average concentration was 127 ng mL^−1^. Tylosin was present at low concentrations in all samples, with the highest average concentration of 45 ng mL^−1^ in chicken manure from Kiambu.

The level of full statistical significance was set at α = 0.05. Three-way ANOVA generally showed that the livestock group (cow, pig, or chicken) was not a significant factor explaining differences in antibiotic concentrations of the manure and soil samples. Only for sulfadimethoxine and oxytetracycline did this factor show marginal significance (α ≤ 0.1), with p-values of 0.090 and 0.100, respectively. The sample type (manure or soil) also had no significant effect on the antibiotic concentration. Only tetracycline showed a marginal significance with a p-value of 0.078, indicating a small influence of the sample type on the residue levels. Finally, analysis of the county (Machakos, Kiambu and Kajiado) showed that only for oxytetracycline were differences significant (p = 0.042). Two-way variations were tested but interactions of livestock group and sample type were not significant for any of the antibiotics; only oxytetracycline revealed marginal significance (p = 0.090). The interaction between livestock group and county was statistically significant only for sulfadimethoxine (p = 0.044), while the interaction between sample type and county had no significant effect. Three-way interactions (livestock group, sample type and county) were significant for oxytetracycline (p = 0.045) and marginally significant for sulfadimethoxine (p = 0.055; Table 1).

### Abundance of antimicrobial resistance genes

**Fig 3:**
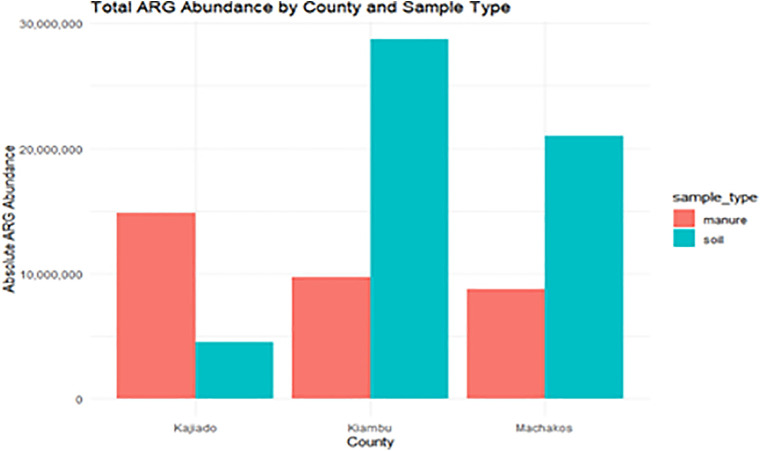
Total abundance of ARGs in manure and soil samples from the three different counties.

In Kajiado county, manure samples showed a substantially higher ARG abundance as compared to soil samples. Conversely, Kiambu and Machakos exhibited substantially higher levels of ARG abundance in the soil samples (Fig 3).

In terms of specific genes, the *aadA* gene showed the highest abundance among all the ARGs analysed particularly in Machakos which suggest possible high usage of aminoglycosides in the area. The *ermB* gene was also present in high values in the same county. Sul1 which is associated with sulphonamide resistance, showed a considerable variation.

**Fig 4:**
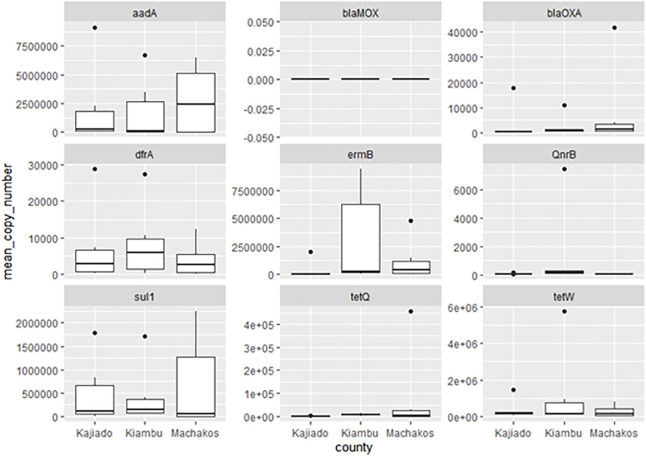
Distribution of mean copy numbers for selected antibiotic resistance genes (ARG) in manure and soil samples from the three counties in Kenya.

On the other hand, *blaMOX* and *QnrB* displayed a very low abundance across all counties. *tetW* and *tetQ* genes, which are both tetracycline resistance genes showed moderate levels with slightly higher copy numbers in Machakos. As shown in Fig 4, there is a distinct spatial heterogeneity in ARG distribution, with higher levels of several key resistance genes like *aadA, sul1*and *blaOXA* generally observed in samples from Machakos*.*

### Relative quantification

**Fig 5:**
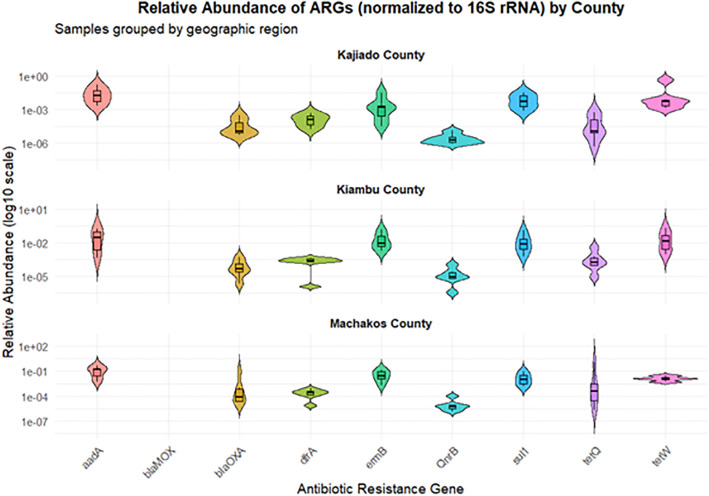
Relative abundance of antibiotic resistance genes (ARGs) across all samples, separated according to the three counties from which they originate, and normalised to the 16S rRNA gene copy numbers (ARG/16S).

The relative abundance of each ARG was obtained by dividing its absolute abundance by the abundance of the 16S rRNA genes in the same sample. This provided a standardised measure of gene prevalence within each of the samples tested. The findings of this study showed that in Kajiado County, *aadA*, *ermB*, and *tetW* genes exhibited the highest abundance levels while *blaOXA and sul1* were relatively low. In Kiambu County, there was a general reduction in abundance with a measurable presence of *ermB* and *QnrB.* Machakos County also showed similar patterns but with *aadA* and *tetW* showing higher levels as shown in Fig 5.

**Fig 6:**
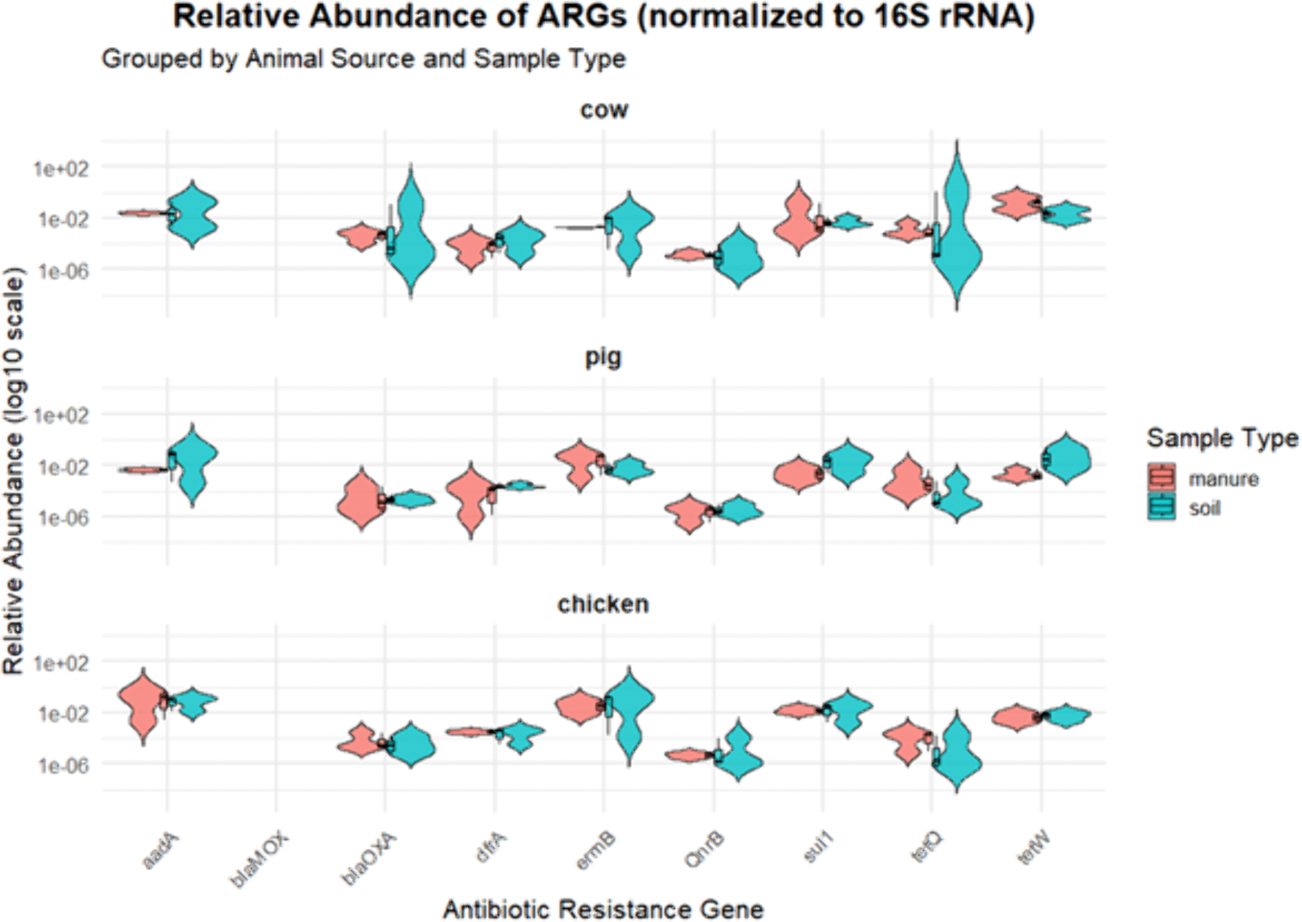
Relative abundance of ARGs (normalized to 16S rRNA gene abundance) in manure and soil samples, grouped according to the livestock group from which the manure originated.

The relative abundance of the ARGs was analysed with regard to the sample type (manure or soil) and the livestock group (cattle, pig and chicken). In the samples from cattle, the *aadA, ermB, sul1* and *tetQ* were more abundant in manure compared to soil samples. The soil samples retained detectable levels of *ermB* and *tetQ* ARGs*,* whereas the *blaMOX* and *blaOXA* ARGs exhibited lower abundance. In the pig samples, *ermB, tetQ* and *tetW* were particularly prevalent in manure compared to soil samples, which showed a reduced ARG abundance. However, *ermB* and *qnrB* had consistently high abundance, while *blaOXA* and *sul1* displayed low to moderate abundance across both sample types. In chicken samples, *aadA, ermB* and *tetQ* were dominant in both manure and soil samples with a higher variability in manure. *blaOXA* and *qnrB* showed low abundance in all samples (Fig 6)

### Factors affecting the antibiotic resistance gene pattern in the agricultural samples

**Fig 7:**
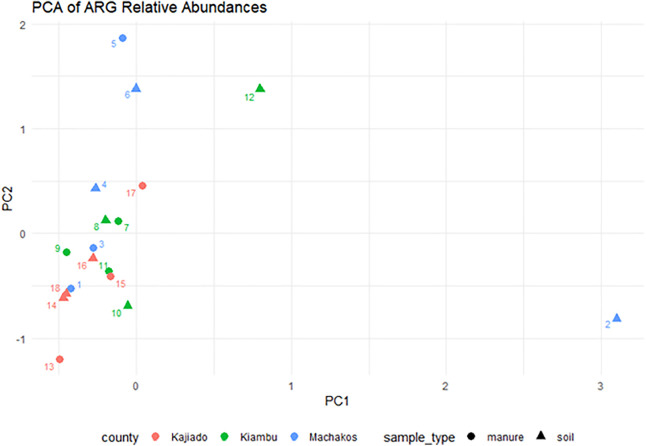
Principal component analysis (PCA) plot of the antibiotic resistance gene ARGs) patterns in manure and soil samples from three counties (Kajiado, Kiambu and Machakos).

Principal component analysis revealed that most samples shared similar ARG profiles. Most samples were tightly clustered near the point of origin (Fig 7) which suggest a similar composition among those samples. However, some samples were clearly distinct due to the environmental and biological factors.

The clustering patterns indicate that most of the samples, and particularly those from Kajiado, are tightly grouped near the origin. By contrast, samples from Machakos and Kiambu are more dispersed across both axes, with the soil samples 2 and 12 and also 5 and 6 clearly deviating from the others. The soil samples were more widely separated in the PCA space than the manure samples.

**Fig 8:**
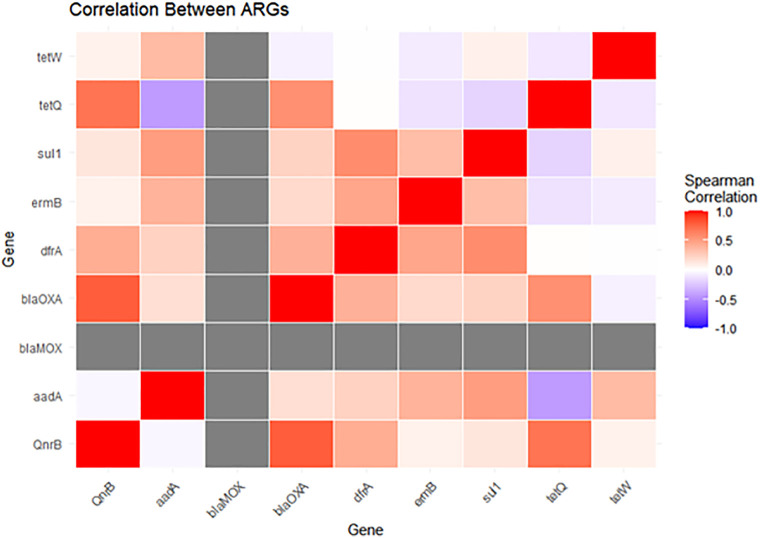
Spearman correlation coefficient between the abundance of different antibiotic-resistance genes in the samples examined.

Spearman correlation revealed that the ARGs *ermB* and *sul1*, *aadA* and *QnrB* and *tetQ* and *tetW* were strongly and positively correlated. As shown in the heatmap (Fig 8), the abundance of a few ARGs such as between *tetQ* and *aadA* was negatively correlated, which is probably due to different distributions or regulatory patterns.

## Discussion

The spatial analysis of antibiotic residues in manure and soil samples from three counties in Kenya revealed a significant disparity among the counties, with the highest antibiotic concentrations measured in Kiambu County, followed by Machakos and Kajiado counties. Tetracyclines were observed to be the most frequently occurring class of antibiotics, followed by enrofloxacin (fluoroquinolone) and tylosin (macrolide). These findings are supported by a previous study conducted among poultry farmers in Kiambu county that showed a widespread, over-the-counter purchase and use of antibiotics [37]. It was consistently reported that the use of tetracyclines (34%) was higher than that of sulphonamides and tylosin (10–15%) [37]. Among the antibiotics tested in our study, tetracyclines (especially oxytetracycline) were the most dominant across all samples, with concentrations higher than 1000 ng mL^−1^ in pig manure from Kiambu. Oxytetracycline was widely used in animal husbandry, with over 81% of cattle farmers reporting its use [38]. Tetracyclines have been identified as environmental pollutants worldwide [39], and residues of tetracycline antibiotics have been found in agricultural soils and vegetables at levels as high as 171.07–660.20 μg kg^−1^ for tetracycline, 25.38–345.78 μg kg^−1^ for oxytetracycline, and 170.77–707.47 μg kg^−1^ for chlortetracycline [40]. However, concentrations of sulphonamides and macrolides were low or even undetectable. It is reasonable to assume that this is due, on the one hand, to the frequency of use mentioned above and, on the other hand, to the fact that sulphonamides are more rapidly degraded or leached from soil compared to other antibiotics [41,42].

Some antibiotics, such as sulfadimethoxine and amoxicillin, were found only in some of the composite samples, whereas the antibiotics tetracycline, oxytetracycline and the fluoroquinolone enrofloxacin were detected in all samples. A prerequisite of the relatively high frequency and concentration of tetracycline residues and prevalence of the associated *tetQ* and *tetW* ARGs in both manure and soil samples is partly due to the fact that tetracyclines are hardly metabolized in the digestive systems of humans and animals. Between 50–80% of the molecules are excreted and subsequently released into the environment, where they persist [43]. However, high excretion rates and persistence in soil are also observed with other antibiotics [44,45] The consistent and high levels of contamination in the samples therefore serve as evidence of the widespread use of several tetracyclines and fluoroquinolones in the Kenyan counties studied. Manure samples, especially from pig, consistently showed high residue levels, emphasizing the risk posed by untreated or poorly managed animal waste. Interestingly, in several cases, soil samples showed concentrations comparable to, or even higher than, those in the corresponding slurry samples. Given that composite samples were analysed, this suggests in particular that antibiotic residues may accumulate over time. These findings emphasize the environmental persistence of certain antibiotics and the need to assess their fate in soil, especially when organic fertilizers are used repeatedly.

These antibiotic residues in the environment pose a significant risk to the ecosystem stability and human health by disrupting biological processes across multiple trophic levels. Antibiotic residues in soil can alter the structure and function of microbial communities, inhibit plant growth, and affect soil faunal species [46]. In soil ecosystems, antibiotics can substantially interfere with enzymatic activities that are crucial for nutrient cycling and overall soil functionality. For instance, sulphonamides have been shown to reduce the activity of key soil enzymes like urease and dehydrogenase, thereby impairing nitrogen cycling and soil fertility and potentially leading to long-term deterioration in soil quality [47,48]. This can lead, not least, to plants being adversely affected both directly by the antibiotics and indirectly by their negative impact on the nutrient cycle in the soil [49,50].

The absolute and relative quantification of ARGs showed that the spread of antibiotics in slurry and soil is accompanied by an increase in ARGs. This was the case in all three districts studied. Kiambu soil samples exhibited the highest ARG leads with up to 30 million copies per gram, whereas Kajiado manure samples contained markedly more ARGs than the corresponding soils, which highlights the county-specific accumulation and pattern of ARGs. Among the ARGs analysed, *aadA* (aminoglycoside resistance), *ermB* (macrolide resistance), and *sul1* (sulphonamide resistance) showed the highest absolute abundances, particularly in samples from Machakos and Kiambu. This reflects both the prevalence and selective pressure of the corresponding antibiotics in these regions. The detection of *tetQ* and *tetW* genes that confer resistance of tetracycline further mirrored the chemical residue data even though only composite samples were analysed. These genes were particularly prevalent in samples from Machakos county, underscoring the consistent relationship between tetracycline residue load and associated resistome. In contrast, *qnrB* (quinolone resistance) was detected only rarely, and *blaMOX* (beta-lactam resistance) was not detected. This indicated either limited exposure to these classes of antibiotics or low environmental persistence of their genetic determinants.

Normalization of ARG abundance to *16S rRNA* gene copy number provided a clearer view of ARG prevalence independent of the microbial biomass. Strikingly, *tetQ* approached a 1:1 ratio with *16S rRNA* in some samples. This may be indicative of intense selective pressure in certain microenvironments. The other genes *aadA, ermB* and *dfrA* showed moderate to high relative abundance, which suggested a widespread dissemination across the microbial communities. Hierarchical clustering and heatmap analysis confirmed that *tetQ* and *tetW* were the most prevalent and co-occurring genes across samples. Principal component analysis (PCA) further demonstrated clustering of most samples, indicating similarity in ARG profiles, whereas only a few samples exhibited a distinct resistome composition.

Spearman correlation analysis revealed several strong positive relationships, for example between *ermB* and *sul1*, *aadA* and *qnr***,** and *tetQ* and *tetW.* These findings may suggest that co-selection and horizontal gene transfer may have shaped ARG co-occurrence; however, this may have been affected by the use of composite samples. This makes it all the more important to highlight the negative correlations that were also found, such as between *tetQ* and *aadA*. These may reflect ecological trade-offs or differing niches among the resistant microbial communities.

This is the first study of antibiotic residues and the prevalence of antibiotic-resistant genes in the agricultural environment in Kenya. Due to the feasibility of the study and in order to examine an initial representative sample set, the study was focused on only three of Kenya’s 47 counties. Additionally, it was not possible to obtain comprehensive data on farmers’ use of antibiotics, restricting the ability to fully contextualize the findings. A total of 180 individual samples were collected and subsequently pooled into 18 samples, depending on sample origin (county) and type of livestock. It should be noted once again that on the one hand pooling contributed to a loss of resolution at the individual sample level but on the other hand eliminated case specific extremes and mismatches of samples.

## Conclusion

This study demonstrates a clear spatial heterogeneity in antibiotic residues and antibiotic residue distribution and antibiotic resistance genes (ARG) prevalence across Machakos, Kiambu and Kajiado Counties, Kenya. It revealed a significant link between antibiotic use practices and environmental contamination. Kiambu County showed the highest antibiotic residues loads, especially of tetracyclines like oxytetracycline, corroborating prior evidence of intensive and mostly unregulated use in livestock farming. The high concentrations observed in manure, particularly from pigs, and the elevated residue levels in soils indicate that antibiotics persist and accumulate in the soil environment. The persistence will contribute to a sustained selective pressure on environmental microbial communities, thereby promoting the proliferation of ARGs.

Resistome analysis is suited to show a widespread presence of ARGs in the three counties with some notable county-specific variation ion ARGs’ abundance and patterns. The strong alignment between the residue data and the prevalence of associated resistance genes selection highlights the ecological linkage between antibiotic contamination and genetic resistance selection. Particularly concerning is the near 1:1 ratio of *tetQ* to 16S rRNA gene copies in certain samples, suggesting pervasive tetracycline resistance and intense selective pressure within microbial populations. The positive correlations among multiple ARGs further indicate that co-selection and horizontal gene transfer are actively shaping ARG co-occurrence and dissemination patterns. Collectively these findings underscore the role of animal production systems as significant reservoirs and amplifiers of antibiotic resistance within the environment.

Given the findings of this study, we recommend strengthening the regulation and stewardship of antibiotics in livestock production, including strict prescription-only antibiotic sales and stringent monitoring of antibiotics usage in animal husbandry. Additional treatment of livestock manure such as composting or anaerobic digestion before use in crop production can lower antibiotic residues and ARG loads. There is need for the government through the ministry of agriculture and the ministry of environment to establish a systematic national surveillance framework from tracking antibiotic residues and ARGs across environmental matrices like manure, soil water etc. There is need for integrated efforts using the one health approach that combines environmental, agriculture and health policies in order to mitigate the environmental degradation due to overuse and misuse of antibiotics. We further recommend conducting a cross-sectional study encompassing a broader geographical scope beyond the three counties examined, with sampling performed during both the wet and dry seasons to enable robust seasonal comparisons. Future investigations should also incorporate additional environmental determinants known to influence ARG abundance, including soil pH, heavy metal concentrations, and other physicochemical parameters that may contribute to ARG persistence and selection in agricultural soils.

## Supporting information

S1 File Table showing primer information, amplicon sequences and NCBI reference sequences used for 16s rRNA genotyping and PCR amplification of antibiotic resistance genes associated with β-lactam, aminoglycoside, tetracycline, fluoroquinolone, and trimethoprim resistance. (DOCX)

S2 File Raw concentration data (ng ML^−1^) for veterinary antibiotics residues quantified from manure and soil samples from livestock production systems in Machakos, Kiambu and Kajiado, including sample metadata and analytical replicates. (XLSX)
