# Supplementary material for: Co-occurrence of antibiotic residues and Antimicrobial resistance genes in animal manure and agricultural soils from Machakos, Kiambu, and Kajiado Counties, Kenya
Source: PLoS One. 2026 Jul 24;21(7):e0337311. doi: 10.1371/journal.pone.0337311 (PMC13399453; doi:10.1371/journal.pone.0337311)
Supplement: S1 File — (DOCX) [file pone.0337311.s001.docx]

Supplementary material 1: primer and amplicon sequence

| **16 s rRNA Genotyping** | | |  |  |  | | |
| --- | --- | --- | --- | --- | --- | --- | --- |
| **Gene** | | **Primer**  **-ID** | **Primer Sequence** |  |  | | |
| 16s rRNA | | 515-F | GTGCCAGCMGCCGCGGTAA |  |  |  |  |
|  |  | 806-R | GGACTACHVGGGTWTCTAAT |  |  |  |  |
| **Resistance profiles** | | | | | | | |
| **Antibiotic** | **Gene target** | **Primer ID** | **Primer Sequence** | **Amplicon sequence** | **NCBI Reference Sequence** | | |
| Β-lactams | *bla*MOX -5 | blaMOX -5-F | ACCAGCTCGGCGGATCTG | \| 137 to base 405 \| \| --- \|   > 269 bp CMY-1/MOX family class C beta-lactamase, base  ( R - F).  GAGCCGGTCTTGTTGAAGAGCACAGGGT  GCCCTGTTGCAGCGGGCGCGGGCGCCGC  CGGGTTCGCATTGTAAATCATCGCCGGG  GAGTTGCCCGCCAGCAGCGTCTGCTCGC  TGACGGGATAGGGGTAACGCTCCCAGCC  CAGTCCCTGGGTCATCTCCCCCACCGCA  TACTGGCCCTGGTGAGTCAGATCGATGG  CCTGCTGCATGGCCGCATTATCCACCCC  GCTGATGTTCGCCTTCACAAAGCGCAGC  AGATCCGCCGAGCTGGT |  | **>NG_074725.1** | *Aeromonas caviae* BP25m blaMOX gene  /MOX family class C beta-lactamase MOX  ATGCAACAACGACAATCCATCCTGTGGGGCGCTCTGGCCACCCTGA  GTGACAAGGCGGCGACCGATCC  CCTGCGCCCCGTGGTGGATGCCAGCATCCGGCCGCTGCTCAAGGAG  CAGGATCCCGGGCATGGCGGTGGCCGTGCTCAAGGATGGCAAGG CCCACTATTTCAACTACGGTGTGGCCGATCGGGAGCGCGCAGTCGG  TGTCAGCGAGCAGACCCTGTTCGAGATAGGCTCCGTGAGCAAGACC  CCGCGACGCTGGGGGCCTACGCCGTGGTGCAGGGGGGCTTCG AGCTCGATGACAAGGCGAGTCTGTTCGCCCCCTGGCTCAAGGGATC  CGCCTTTGACAACATCACCATGGGGGAGCTGGCTACCTACAGCGCG  TTGCCGCTGCAATTCCCCGAGGAGGTGGATTCGCTCGAGA AGATGCAGGCCTACTACCGCCAGTGGACCCCAGCCTACTCGCCGGG  TTCCCATCGCCAGTACGCCAACCCCAGCATCGGGCTCTTTGGCTAT  GAGCAGCATGAAGCAGCCGTTCGATCGCCTGATGGAGC AGACGATCCTGCCGGGGCTTGGCCTGTACCATACCTACCTCACTGT  GCCCGAGCAGGCCATGGGGCACTACGCCTACGGCTACTCGAAGGAG  TCCGCGTCACTCCCGGCATGCTGGCGGACGAGGCCT  ACGGCATCAAGACCAGCTCGGCGGATCTGCTGCGCTTTGTGAAGGC  ACATCAGCGGGGTGGATAATGCGGCCATGCAGCAGGCCATCGAC  GGCCAGTATGCGGTGGGGGAGATGACCCAGGGAC TGGGCTGGGAGCGTTACGCCTATCCCGTCAGCGAGCAGACGCTGCT  GGCGGGCAACTCCCCGGCGATGATTTACAATGCCAACCCGGCGGCG  AACAGGGCACCCTGTGCTCTTCAACAAGACCG GCTCGACCAACGGCTTCGGGGCCTATGTGGCCTTCGTGCCGGCCAA  AGGGATTGGCATCGTCATGCTGGCCAATCGCAACTACCCCAACGAG  CCCACGCCATCCTGACGAAACTGGCCAGGT |
|  |  |  |  |  |  | for CMY-1 15, complete CDS TGTGGGCCGGTCTGGCTCATGCCG  CA  CTGA  GGCGGC  CTGGCGGC  GACAAGCCCA  GA  CTGACTCACCAG  CCCGCGCCCGCTGC  GCGCGCATCAAGGCGG  AA |  |
|  |  | blaMOX -5-R | GAGCCGGTCTTGTTGAAGAGC |  |  |  |  |

|  | *bla*OXA | blaOXA -F | AGGCACGATAGTTGTGGCAGA  C | >240 Class-D beta lactamase, base 123 to base 362 (F -  R) AGGCACGATAGTTGTGGCAGACGAACGC  CAAGCGGATCGTGCCATGTTGGTTTTTG  ATCCTGTGCGATCGAAGAAACGCTACTC  GCCTGCATCGACATTCAAGATACCTCAT  ACACTTTTTGCACTTGATGCAGGCGCTG  TTCGTGATGAGTTCCAGATTTTTCGATG  GGACGGCGTTAACAGGGGCTTTGCAGGC  CACAATCAAGACCAAGATTTGCGATCAG  CAATGCGGAATTCTAC |  | **>NG_049612.1** | | :101-928 *Pseudomonas aeruginosa* | |
| --- | --- | --- | --- | --- | --- | --- | --- | --- | --- |
|  |  |  |  |  |  | blaOXA gene for OXA-2 family extended-spectrum | | |  |
|  |  |  |  |  |  | class D beta-lactamase OXA-32, complete CDS ATGGCAATCCGAATCTTCGCGATACTTTTCTCCATTTTTTCTCTTG CCACTTTCGCGCATGCGCAAGAAGGCACGCTAGAACGTTCTGACTG  GAGGAAGTTTTTCAGCGAATTTCAAGCCAAAGGCACGATAGTTGTG GCAGACGAACGCCAAGCGGATCGTGCCATGTTGGTTTTTGATCCTG TGCGATCGAAGAAACGCTACTCGCCTGCATCGACATTCAAGATACC TCATACACTTTTTGCACTTGATGCAGGCGCTGTTCGTGATGAGTTC  CAGATTTTTCGATGGGACGGCGTTAACAGGGGCTTTGCAGGCCACA ATCAAGACCAAGATTTGCGATCAGCAATGCGGAATTCTACTGTTTG GGTGTATGAGCTATTTGCAAAGGAAATTGGTGATGACAAAGCTCGG  CGCTATTTGAAGAAAATCGACTATGGCAACGCCGATCCTTCGACAA GTAATGGCGATTACTGGATAGAAGGCAGCATTGCAATCTCGGCGCA GGAGCAAATTGCATTTCTCAGGAAGCTCTATCGTAACGAGCTGCCC TTTCGGGTAGAACATCAGCGCTTGGTCAAGGATCTCATGATTGTGG  AAGCCGGTCGCAACTGGATACTGCGTGCAAAGACGGGCTGGGAAGG CCGTATGGGTTGGTGGGTAGGATGGGTTGAGTGGCCGACTGGCTCC GTATTCTTCGCACTGAATATTGATACGCCAAACAGAATGGATGATC  TTTTCAAGAGGGAGGCAATCGTGCGGGCAATCCTTCGCTCTATTGA  AGCGTTACCGCCCAACCCGGCAGTCAACTCGGACGCAGCGCGATAA | | |  |
|  |  | blaOXA -R | GTAGAATTCCGCATTGCTGAT  CGC |  |  |  |  |  |  |
|  | *bla*TEM | blaTEM -F | GAACCGGAGCTGAATGAAGCC | \| 764 \| \| --- \|   >275 TEM FAMILY Class A Beta lactamase, base 490 to base  (F - R)  GAACCGGAGCTGAATGAAGCCATACCAA  ACGACGAGCGTGACACCACGATGCCTGC  AGCAATGGCAACAACGTTGCGCAAACTA  TTAACTGGCGAACTACTTACTCTAGCTT  CCCGGCAACAATTAATAGACTGGATGGA  GGCGGATAAAGTTGCAGGACCACTTCTG  CGCTCGGCCCTTCCGGCTGGCTGGTTTA  TTGCTGATAAATCTGGAGCCGGTGAGCG  TGGATCTCGCGGTATCATTGCAGCACTG  GGGCCAGATGGTAAGCCCTCCCG |  | **>KP686109.1** | *Escherichia coli* strain PALTEM137b | | |
|  |  |  |  |  |  | extended spectrum beta-lactamase enzyme (TEM) | | |  |
|  |  | *Bla*TEM  -R | CGGGAGGGCTTACCATCTGG |  |  | gene, partial cds  ATGAGTATTCAACATTTTCGTGTCGCCCTTATTCCCTTTTTTGCGG CATTTTGCCTTCCTGTTTTTGCTCACCCAGAAACGCTGGTGAAAGT AAAAGATGCTGAAGATCAGTTGGGTGCACGAGTGGGTTACATCGAA  CTGGATCTCAACAGCGGTAAGATCCTTGAGAGTTTTCGCCCCGAAG AACGTTTTCCAATGATGAGCACTTTTAAAGTTCTGCTATGTGGTGC GGTATTATCCCGTGTTGACGCCGGGCAAGAGCAACTCGGTCGCCGC  ATACACTATTCTCAGAATGACTTGGTTGAGTACTCACCAGTCACAG AAAAGCATCTTACGGATGGCATGACAGTAAGAGAATTATGCAGTGC  TGCCATAACCATGAGTGATAACACTGCGGCCAACTTACTTCTGACA ACGATCGGAGGACCGAAGGAGCTAACCGCTTTTTTGCACAACATGG GGGATCATGTAACCCGCCTTGATAGTTGGGAACCGGAGCTGAATGA AGCCATACCAAACGACGAGCGTGACACCACGATGCCTGCAGCAATG GCAACAACGTTGCGCAAACTATTAACTGGCGAACTACTTACTCTAG  CTTCCCGGCAACAATTAATAGACTGGATGGAGGCGGATAAAGTTGC AGGACCACTTCTGCGCTCGGCCCTTCCGGCTGGCTGGTTTATTGCT | | |  |

|  |  |  |  |  | GATAAATCTGGAGCCGGTGAGCGTGGATCTCGCGGTATCATTGCAG  CACTGGGGCCAGATGGTAAGCCCTCCCGTATCGTAGTTATCTACAC GACGGGGAGTCAGGCAACTATGGATGAACGAAATAGACAGATCGCT  GAGATAGGTGCCTCACTGATTAAGCATT | | | | |
| --- | --- | --- | --- | --- | --- | --- | --- | --- | --- |
| Diaminopyrimidi nes | *dfr*A | dfrA1F | ACATACCCTGGTCCGCGAAAG | >240 trimethoprim-resistant dihydrofolate reductase DfrA14 base 59 to base 298 (F - R).  ACATACCCTGGTCCGCGAAAGGGGAGCA  GCTACTTTTTAAAGCATTGACCTACAAT  CAGTGGCTTCTGGTGGGTCGCAAGACGT  TTGAATCTATGGGCGCACTCCCCAATAG  GAAATACGCGGTCGTTACCCGCTCAGGT  TGGACATCAAATGATGACAATGTAGTTG  TATTTCAGTCAATCGAAGAGGCCATGGA  CAGGCTAGCTGAATTCACCGGTCACGTT  ATAGTGTCTGGTGGCG |  | **>CP172466.1** | :2991849-2992322 *Salmonella*  *enterica subsp. enterica* serovar Agona strain  R24.1396 chromosome, complete genome  TTGAAAGTATCATTGATGGCTGCGAAAGCGAAAAACGGCGTGATTG GTTGCGGTCCAGACATACCCTGGTCCGCGAAAGGGGAGCAGCTACT  TTTTAAAGCATTGACCTACAATCAGTGGCTTCTGGTGGGTCGCAAG  GTTTGAATCTATGGGCGCACTCCCCAATAGGAAATACGCGGTCG TTACCCGCTCAGGTTGGACATCAAATGATGACAATGTAGTTGTATT  TCAGTCAATCGAAGAGGCCATGGACAGGCTAGCTGAATTCACCGGT  TTATAGTGTCTGGTGGCGGAGAAATTTACCGAGAAACATTAC CCATGGCCTCTACGCTCCACTTATCGACGATCGACATCGAGCCAGA  GGGGGATGTTTTCTTCCCGAGTATTCCAAATACCTTCGAAGTTGTT CAACACTTTACTTCAAACATTAACTATTGCTATCAAATTT | | |
|  |  |  |  |  |  | AC  CACG  TTTGAG  GGAAAAAGGGTTAA |  |  |  |
|  |  | dfrA1R | CGCCACCAGACACTATAACGT  GA |  |  |  |  |  |  |
| Fluoroquinolone s | *qnrB* | qnrB23 -F | CGACCTGAGCGGCACTGAATT  TA | \| base 135 to \| \| --- \|   >405 bp qnrB quinolone resistance pentapeptide repeat protein QnrB23, complete CDS,  base 539 (F - R)  CGACCTGAGCGGCACTGAATTTATCGGC  TGTCAGTTCTATGATCGTGAAAGCCAGA  AAGGGTGCAATTTTAGTCGTGCGATGCT  GAAAGATGCCATTTTTAAAAGCTGTGAT  TTATCCATGGCGGATTTTCGCAATGCCA  GTGCGCTTGGCATTGAAATTCGCCACTG  TCGTGCGCAAGGCGCAGATTTCCGCGGC  GCAAGCTTTATGAATATGATCACTACTC  GCACCTGGTTTTGCAGCGCATATATCAC  TAACACAAATCTAAGCTACGCCAATTTT  TCGAAAGTCGTGCTGGAAAAGTGTGAGC  TGTGGGAAAACCGTTGGATGGGTGCCCA  GGTACTGGGCGCGACGTTCAGTGGTTCA  GATCTCTCCGGCGGCGAGTTTTCGACTT  TCGACTGGCGAGC |  | **>NG_050484.1** | | :37-681 *Citrobacter freundii* S008 | |
|  |  |  |  |  |  | pS008 qnrB gene for quinolone resistance | | |  |
|  |  |  |  |  |  | pentapeptide repeat protein QnrB23, complete  CDS  ATGGCTCTGGCACTCGTTGGCGAAAAAATTGACAGAAACCGCTTCA CCGGTGAGAAAATTGAAAATAGTACATTTTTTTACTGTGATTTTTC AGGTGCCGACCTGAGCGGCACTGAATTTATCGGCTGTCAGTTCTAT  GATCGTGAAAGCCAGAAAGGGTGCAATTTTAGTCGTGCGATGCTGA AAGATGCCATTTTTAAAAGCTGTGATTTATCCATGGCGGATTTTCG CAATGCCAGTGCGCTTGGCATTGAAATTCGCCACTGTCGTGCGCAA GGCGCAGATTTCCGCGGCGCAAGCTTTATGAATATGATCACTACTC GCACCTGGTTTTGCAGCGCATATATCACTAACACAAATCTAAGCTA CGCCAATTTTTCGAAAGTCGTGCTGGAAAAGTGTGAGCTGTGGGAA  AACCGTTGGATGGGTGCCCAGGTACTGGGCGCGACGTTCAGTGGTT CAGATCTCTCCGGCGGCGAGTTTTCGACTTTCGACTGGCGAGCAGC AAACTTCACACATTGCGATCTGACCAATTCGGAGTTGGGGGACTTA GATATTCGGGGCGTTGATTTACAAGGCGTTAAGTTGGACAACTACC  AGGCATCGTTGCTCATGGAACGTCTTGGCATCGCGATTATTGGCTA  G | | |  |
|  |  | qnrB23 -R | GCTCGCCAGTCGAAAGTCGAA |  |  |  |  |  |  |

|  | *tetQ* | tetQ-F | TGGATTGAAGACCCGTCTTTG  TCC | >438 bp product tetracycline resistance ribosomal protection protein Tet(Q) (tet(Q)) gene, partial cds, base 1108 to base 1545 (F -  R). TGGATTGAAGACCCGTCTTTGTCCTTTT  CCATAAACTCATATAGTGATGAATTGGA  AATCTCGTTATATGGTTTGACACAAAAG  GAAATCATACAGACATTGCTGGAAGAAC  GATTTTCCGTCAAGGTCCATTTTGATGA  GATCAAGACTATCTACAAAGAACGACCT  GTAAAAAAGGTCAATAAGATTATTCAGA  TCGAAGTGCCACCCAACCCTTATTGGGC  CACAATAGGGCTGACTCTTGAACCCTTG  CCGTTAGGGACAGGGTTGCAAATCGAAA  GTGACATCTCCTATGGTTAT  CTGAACCATTCTTTTCAAAATGCCGTTT  TTGAAGGGATTCGTATGTCTTGCCAATC  TGGTTTACATGGATGGGAAGTGACTGAT  CTGAAAGTAACTTTTACTCAAGCCGAGT  ATTATAGCCCGGTAAGTACACCTGCT |  | **>MT050499.1** | Uncultured bacterium clone | | |
| --- | --- | --- | --- | --- | --- | --- | --- | --- | --- |
|  |  |  |  |  |  | tet(Q)_4_NHP_NGR_2017 tetracycline resistance | | |  |
|  |  |  |  |  |  | ribosomal protection protein Tet(Q) (tet(Q)) gene, partial cds  ATGAATATTACAAATTTAGGAATTCTTGCTCACATTGATGCAGGAA AAACTTCCGTAACCGAGAATCTGCTGTTTGCCAGTGGAGCAACGGA AAAGTGCGGCCGTGTGGATAATGGTGACACCATAACAGACTCTATG  GATATAGAGAAACGTAGAGGAATTACTGTTCGGGCTTCTACGACAT CTATTATCTGGAATGGAGTGAAATGCAATATCATTGACACTCCGGG ACACATGGATTTTATTGCGGAAGTGGAGCGGACATTCAAAATGCTT  GATGGAGCAGTCCTCATCTTATCCGCAAAGGAAGGCATACAAGCGC AAACAAAGTTGCTGTTCAATACTTTACAAAAACTGCAAATCCCGAC AATTATATTTATCAATAAAATTGACCGTGACGGTGTGAATTTAGAG  CGTTTGTATCTGGATATAAAAACAAATCTGTCTCAAGATGTCCTGT TTATGCAAACTATTGTCGATGGATTGGTTTATCCGATTTGCTCCCA AACATATATAAAGGAAGAATACAAAGAATTTGTATGCAACCATGAC GACAATATATTAGAACGATATTTGGCGGATAGCGAAATTTCACCGG  CTGATTATTGGAATACGATAATCGATCTTGTGGCAAAAGCCAAAGT  CTATCCGGTACTACATGGATCAGCAATGTTCAATATCGGTATCAAT GAGTTGTTGGACGCCATCTCTTCTTTTATACTTCCTCCAGAATCAG  TCTCAAACAGACTTTCAGCTTATCTCTATAAGATAGAGCATGACCC CAAAGGACATAAAAGAAGTTTTCTAAAAATAATTGACGGAAGTCTG AGACTTCGAGACATTGTAAGAATCAACGATTCGGAAAAATTCATCA  AGATTAAAAATCTAAAGACTATTTATCAGGGCAGAGAGATAAATGT TGATGAAGTGGGGGCCAATGATATCGCGATTGTAGAAGATATGGAA GATTTTCGAATCGGAGATTATTTAGGTGCTAAACCTTGTTTGATTC  AAGGGTTATCTCGTCAGCATCCCGCTCTCAAATCCTCCGTCCGGCC AGACAGGCCCGAAGAGAGAAGCAAGGTGATATCCGCTCTGAATACA TTGTGGATTGAAGACCCGTCTTTGTCCTTTTCCATAAACTCATATA  GTGATGAATTGGAAATCTCGTTATATGGTTTGACACAAAAGGAAAT CATACAGACATTGCTGGAAGAACGATTTTCCGTCAAGGTCCATTTT GATGAGATCAAGACTATCTACAAAGAACGACCTGTAAAAAAGGTCA  ATAAGATTATTCAGATCGAAGTGCCACCCAACCCTTATTGGGCCAC AATAGGGCTGACTCTTGAACCCTTGCCGTTAGGGACAGGGTTGCAA ATCGAAAGTGACATCTCCTATGGTTATCTGAACCATTCTTTTCAAA  ATGCCGTTTTTGAAGGGATTCGTATGTCTTGCCAATCTGGTTTACA TGGATGGGAAGTGACTGATCTGAAAGTAACTTTTACTCAAGCCGAG TATTATAGCCCGGTAAGTACACCTGCTGATTTCAGACAGCTGACCC CTTATGTCTTCAGGCTGGCCTTGCAACAGTCAGGTGTGGACATTCT | | |  |
|  |  | tetQ-R | AGCAGGTGTACTTACCGGGCT  ATA |  |  |  |  |  |  |
|  |  |  |  |  | CGAACCGATGCTCTATTTTGAGTTGCAGATACCCCAAGCGGCAAGT TCCAAAGCTATTACAGATTTGCAAAAAATGATGTCTGAGATTGAAG ACATCAGTTGCAATAATGAGTGGTGTCATATTAAAGGGAAAGTTCC  ATTAAATACAAGTAAAGACTACGCCTCAGAAGTAAGT | | | | |
| Aminoglycosides | *aadA* | aadA-F | GCCTCTTACACATGGTCAAT | >441 bp aadD1 gene for aminoglycoside Onucleotidyltransferase  ANT(4')-Ia, base 321 to base  761 ( F - R)  CTCTATTTTGCCGATTTATGATTCAGGT  GGATACTTAGAGAAAGTGTATCAAACTG  CTAAATCGGTAGAAGCCCAAACGTTCCA  CGATGCGATTTGTGCCCTTATCGTAGAA  GAGCTGTTTGAATATGCAGGCAAATGGC  GTAATATTCGTGTGCAAGGACCGACAAC  ATTTCTACCATCCTTGACTGTACAGGTA  GCAATGGCAGGTGCCATGTTGATTGGTC  TGCATCATCGCATCTGTTATACGACGAG  CGCTTCGGTCTTAACTGAAGCAGTTAAG  CAATCAGATCTTCCTTCAGGTTATGACC  ATCTGTGCCAGTTCGTAATGTCTGGTCA  ACTTTCCGACTCTGAGAAACTTCTGGAA  TCGCTAGAGAATTTCTGGAATGGGATTC  AGGAGTGGACAGAACGACACGGATATAT  AGTGGATGTGTCAAAACGCAT |  | >NG_047375.1 | | :101-871 Staphylococcus aureus | |
|  |  | aadA-R | ATGCGTTTTGACACATCCACT |  |  | HUC19 pUB110 aadD1 gene for aminoglycoside O- | | |  |
|  |  |  |  |  |  | nucleotidyltransferase ANT(4')-Ia, complete  CDS  ATGAGAATAGTGAATGGACCAATAATAATGACTAGAGAAGAAAGAA TGAAGATTGTTCATGAAATTAAGGAACGAATATTGGATAAATATGG GGATGATGTTAAGGCTATTGGTGTTTATGGCTCTCTTGGTCGTCAG  ACTGATGGGCCCTATTCGGATATTGAGATGATGTGTGTCATGTCAA CAGAAGAAGCAGAGTTCAGCCATGAATGGACAACCGGTGAGTGGAA GGTGGAAGTGAATTTTGATAGCGAAGAGATTCTACTAGATTATGCA  TCTCAGGTGGAATCAGATTGGCCTCTTACACATGGTCAATTTTTCT CTATTTTGCCGATTTATGATTCAGGTGGATACTTAGAGAAAGTGTA TCAAACTGCTAAATCGGTAGAAGCCCAAACGTTCCACGATGCGATT  TGTGCCCTTATCGTAGAAGAGCTGTTTGAATATGCAGGCAAATGGC GTAATATTCGTGTGCAAGGACCGACAACATTTCTACCATCCTTGAC  TGTACAGGTAGCAATGGCAGGTGCCATGTTGATTGGTCTGCATCAT  CGCATCTGTTATACGACGAGCGCTTCGGTCTTAACTGAAGCAGTTA  AGCAATCAGATCTTCCTTCAGGTTATGACCATCTGTGCCAGTTCGT AATGTCTGGTCAACTTTCCGACTCTGAGAAACTTCTGGAATCGCTA GAGAATTTCTGGAATGGGATTCAGGAGTGGACAGAACGACACGGAT  ATATAGTGGATGTGTCAAAACGCATACCATTTTGA | | |  |

Reference

Gibson, C., Kraemer, S. A., Klimova, N., Guo, B., & Frigon, D. (2023). Antibiotic resistance gene sequencing is necessary to reveal the complex dynamics of immigration from sewers to activated sludge. *Frontiers in*

*Microbiology*, *14*(April), 1–13. https://doi.org/10.3389/fmicb.2023.1155956
