# Supplementary material for: Co-occurrence of antibiotic residues and Antimicrobial resistance genes in animal manure and agricultural soils from Machakos, Kiambu, and Kajiado Counties, Kenya
Source: PLoS One. 2026 Jul 24;21(7):e0337311. doi: 10.1371/journal.pone.0337311 (PMC13399453; doi:10.1371/journal.pone.0337311)
Supplement: Attachment — Submitted filename: Response_to_reviewers_auresp_3.docx [file pone.0337311.s006.docx]

Dear Editor,

Please find the responses to the various issues raised by the reviewers.

PONE-D-25-59999

Title: **Co-occurrence of Antibiotic Residues and Antimicrobial Resistance Genes in Animal Manure and Agricultural Soils from Machakos, Kiambu, and Kajiado Counties, Kenya**

PLOS ONE

|  | **Reviewers’ comments** | **Authors response** |
| --- | --- | --- |
|  | **Reviewer 1** |  |
|  | The study design has significant limitations in terms of representativeness and analytical robustness. Convenience  sampling, based on the discretion of farm owners, introduces potential selection bias and limits generalizability. Pooling  180 individual samples into 18 composites further reduces data resolution, obscures variability, and weakens the  validity of subsequent statistical analyses | The limitations of the pooling of samples have been acknowledged in page 6 line 8-20. |
|  | The statistical approach needs clarification and more cautious interpretation. Several results labeled as “marginally  significant” (p-values between 0.05 and 0.10) are overinterpreted. There is no evidence that ANOVA assumptions were  assessed or that multiple-comparison adjustments were made. Given the reduced effective sample size from pooling,  the use of parametric analyses should be more thoroughly justified. | The interpretation of the analysis has been corrected and justified in page 9 line 17-25. |
|  | Some conclusions exceed the study design's support. Attributing ARG distribution to “clear antibiotic use” and  “environmental persistence” is not justified, as there is no direct antibiotic-use data or assessment of temporal  dynamics. The findings indicate associations, not causality. | The justification has been provided in pages 18 and 19 lines 1-23 and 1-5. |
|  | Interpretation of results often relies on presumed antibiotic use patterns, but no primary data on antibiotic usage | Addressed in page 20 line 20-31 |
|  | were collected. This limits the ability to contextualize findings, leading to speculative explanations |  |
|  | Reporting of antibiotic concentrations is inconsistent, with non-standard units for environmental samples and variation across sections. | The concentration harmonized across the manuscript. |
|  | The manuscript would benefit from comprehensive language editing to correct grammatical inconsistencies,  repetition, and unclear sentence structure. | The manuscript has been revised and language corrected |
|  | Methodological details, especially for qPCR and LC-MS/MS analyses, should be expanded to include validation  parameters such as efficiency, recovery, and detection limits. | Revised in page 7line 21-33 |
|  | The discussion section should be more structured to enhance readability and thematic coherence. | Discussion section revised. |
|  | Referencing should be standardized and updated to ensure consistency and alignment with journal formatting  requirements. | References revised. |

Yours Sincerely,

Richard Machoka

Corresponding author.
