# Supplementary material for: Co-occurrence of antibiotic residues and Antimicrobial resistance genes in animal manure and agricultural soils from Machakos, Kiambu, and Kajiado Counties, Kenya
Source: PLoS One. 2026 Jul 24;21(7):e0337311. doi: 10.1371/journal.pone.0337311 (PMC13399453; doi:10.1371/journal.pone.0337311)
Supplement: Attachment — Submitted filename: Response to reviewers.docx [file pone.0337311.s003.docx]

Dear Editor,

Please find the responses to the various issues raised by the reviewers.

PONE-D-25-59999

Title: **Co-occurrence of Antibiotic Residues and Antimicrobial Resistance Genes in Animal Manure and Agricultural Soils from Machakos, Kiambu, and Kajiado Counties, Kenya**

PLOS ONE

|  | **Reviewers’ comments** | **Authors response** |
| --- | --- | --- |
|  | **Reviewer 1** |  |
|  | In page 2, lines 25 - 27 the author does not specify the geographical region that the high concentrations were found, and the reference (Kumar et al 2005) is old | Addressed in page 2 line from line 31 comparative analysis of Thailand, China and Kenya is given. Newer references given |
|  | Lines 25 - 27 of page 2 forms the crux of the study aims, so a more robust literature review is required. | Added a paragraph with additional information from other parts of the world in page 2 line 27-32 and page 3 line 1-7 |
|  | In page 2 lines 23 and 24, it is not clear which category sulphonamides and acetic conjugates fall into. I recommend rewording the sentence to be clearer. It can be written as "some metabolites such as... end up being more potent..." or "some metabolites end up being more potent... while others such as..." Data analysis | Corrected to read “while others such as acetic-conjugated sulphonamides can change back to their parent compounds during manure storage ...” page 2 , line 25-26 |
|  | The alpha value was not defined in the study. | Statistical significance was defined at α = 0.05. Page 12 line 25-26 |
|  | In page 12, line 2 the wrong p value was entered and should be corrected to 0.0547. | Corrected to “nearly significant for sulfadimethoxine (P = 0.055” at page 13, line 6 |
|  | The reference appears to be missing in page 21 lines 15,16 and 17. | Corrected in page 20 line 4 “was higher than that of sulphonamides and tylosin (10-15%) (Kariuki et al., 2023)” |
|  | It would be beneficial for readers to have more information on the specific antibiotic compounds in the background. | Additional paragraph in page 2 line 27-32 to page 3 1-7 that gives more information about antibiotics isolated from manure from different parts of the world. |
|  | Reviewers 2 |  |
|  | a. How was the soil sample collected? b. What type of container was used to collect the soil sample: sterile or nonsterile? c. At what depth of soil was it collected? Was it topsoil or subsoil? | A subsection on sample collection has been added in page 7 line 14-20 that addresses the question |
|  | 1. Statistical Analysis The three-way ANOVA was appropriately applied; however: Some marginally significant values (p = 0.06–0.10) are interpreted as meaningful. These should be described more cautiously. Provide effect sizes to better understand the magnitude of differences. Clarify whether ANOVA assumptions (normality, homogeneity) were checked. | The corrections have been done. The ANOVA assumptions were checked. |
|  | 2. Data Availability The manuscript does not provide full raw datasets (individual sample-level concentrations, Ct values, and standard curves). Recommendation: Upload processed and raw datasets to a public repository (Figshare, OSF, Dryad) to comply with journal policy. | The raw data has been uploaded to Dryad website, although it has not been published due to the fee required, once paid the data will be available under the name Machoka Richard |
|  | 3. Clarity and Organization. The manuscript is generally well written but includes several typographical and grammatical issues. Examples: “the sample was be calculated” is used instead of “the sample size was calculated” (materials and methods) The word "inn" is incorrectly used instead of "in" in the conclusion. "Wo" is used instead of "who" in the acknowledgement section. These represent only a few examples. The manuscript contains many more areas where grammar, phrasing, and overall readability need improvement. Some long paragraphs can be broken into shorter ones to improve readability. | corrected in page 7 line 1 “The sample size was calculated using the following formula” and the other typos were corrected |
|  | 4. Figures and Tables. All figures should include units and error bars where applicable. Supplementary materials should be referenced more consistently. | Corrected |
|  | Clarify whether manure samples were fresh, composted, or mixed; this influences residue degradation kinetics. | Information added at page 7 page line 15 “Sterile spatulas and zip-lock bags were used to collect animal fresh manure” |
|  | 1. How was the soil sample collected? 2. What type of container was used to collect the soil sample, sterile or non-sterile? 3. At what depth of soil was it collected? Was it topsoil or subsoil? There should be a subtitle for sample collection. | Page 6 line 20-25 a paragraph has been added with the answers to the raised questions. |
|  | 6. Interpretation of Results The conclusions are supported by the data; however, there are some limitations to consider: The manuscript occasionally over generalizes findings; for example, attributing all high ARG abundance exclusively to antibiotic pressure without considering co-selection by metals or other stressors. | Corrected, Additional information of other environmental factors that could affect the ARG discussed in page 5 line 7 |
|  | Provide possible explanations for county-specific variations (differences in farming intensity, soil properties, waste handling, etc.). | Provided as a paragraph in page 5 line 7-18 which discusses the differences in the geographical and agricultural practices among the counties |
|  | 7. Literature Context The manuscript cites relevant global and regional literature but could benefit from: The manuscript could benefit from more discussion on environmental drivers of ARG persistence, such as organic matter, soil pH, and manure management practices. The manuscript also needs to incorporate recent studies on soil and manure resistance conducted in Africa between 2019 and 2025. Finally, I recommend Major Revision (Encouraged to Resubmit). The study is strong and worthy of publication but requires improvements in data transparency, polishing of language, and clearer presentation of statistical results. Addressing these issues will enable the manuscript to significantly contribute to AMR research and environmental health in sub-Saharan Africa. | The soil differences in PH, and agricultural practices of the three counties are discussed in page 5 page 7-18. |
|  | Reviewer 3 |  |
|  | 7. Literature Context The manuscript cites relevant global and regional literature but could benefit from: The manuscript could benefit from more discussion on environmental drivers of ARG persistence, such as organic matter, soil pH, and manure management practices. The manuscript also needs to incorporate recent studies on soil and manure resistance conducted in Africa between 2019 and 2025. Finally, I recommend Major Revision (Encouraged to Resubmit). The study is strong and worthy of publication but requires improvements in data transparency, polishing of language, and clearer presentation of statistical results. Addressing these issues will enable the manuscript to significantly contribute to AMR research and environmental health in sub-Saharan Africa. | Agroecological, organic matter composition of soils and agricultural practices have been discussed in page 5 line 7-18  Recent studies added in pages 20 line 4,8,9and 12. |
|  | 2. The sample size calculation was inappropriate for this type of study; the study was exploratory and not inferential. Pooling of the samples into 18 samples alienates on-site differences, and devalues the following three-way ANOVA. The county-level comparisons are not valid because the sampling was pooled, unreplicated, and purposively biased. Also, the statistical software used to calculate the averages, make bar charts, and even perform principal component analysis and other diagrames was not mentioned, nor was the version stated. | The pooling of samples was necessitated by financial inability to analyse all the 180 samples individually. The authors recognize the shortcomings that this presents in the paper and hence the limitation section was added.  Statistical software used stated in page 10 line 25 “.All statistical analysis and data visualization were carried out in R version 4.5.1.  ” |
|  | Minor The abstract states, “A total of 180 samples from 30 farms across the three counties were collected and pooled into 18 samples", The specific type of sample was not stated. There were also grammatical errors, few references for the discussion, and no objective in the introduction, even though it was stated in the abstract. Diagrams are not well labelled to match the in-text writings. No limitation stated and novelty of this paper is unknown. The title and results mention antimicrobial-resistant genes (ARGs) and antibiotic residues, but the extensive literature on the ARGs was not described, which makes the literature review incomplete. | Abstract corrected in page 1 line 20 “A total of 180 animal manure and soil samples from 30 farms across the three counties were collected”  Limitations of the study added at page 7 line 22-27.  The objective of the study stated in page 4 line 7-9 “This study therefore aims at analysing the antibiotic residues and the antimicrobial resistance gene profiles in the three highly agricultural counties in Kenya where antibiotics are routinely used for various livestock species”  Additional information about ARG and how they spread provided in page 3 line 8-22 |
|  | Reviewer 4 |  |
|  | 1. Introduction The introduction is well structured and provides helpful context. To improve clarity, the authors should explicitly state the main objective of the study in the final paragraph. | The main objective stated in page 4 line 7-9 “This study therefore aims at analysing the antibiotic residues and the antimicrobial resistance gene profiles in the three highly agricultural counties in Kenya where antibiotics are routinely used for various livestock species” |
|  | 2. Methods The methods are generally detailed, but several issues require attention: • Sample pooling: The manuscript reports that 180 samples were collected but later pooled into 18 composite samples. The rationale for this approach is not clearly explained. Pooling can substantially reduce variability and may affect the validity of the statistical analysis. • ANOVA assumptions: Three-way ANOVA assumes independent observations. Because the data were pooled, this assumption may not hold. The authors should justify the approach and discuss its limitations. | The limitations of the study has been added in page 7 line 22 onwards. |
|  | 3. Results The results are clearly described and supported by visualizations. I recommend: • Reporting p-values consistently (e.g., to three decimal places) in Table 1. • Ensuring all figures are high-resolution and labeled clearly. | P- value reporting to three decimal places in the table page 13 line 8 onwards |
|  | 4. Discussion The discussion is informative and connects findings to existing literature. However: • Several paragraphs repeat results rather than interpreting them. • The authors could more fully explain potential reasons for county-level differences, such as farming intensity or antibiotic access. | The results section has been reorganized as guided.  The differences in county level practices and soils profiles added in page 5 line 7-18 |
|  | 5. Limitations Important limitations are not fully acknowledged. The authors should consider adding: • The impact of sample pooling on statistical power and interpretation. • The cross-sectional design and lack of temporal or seasonal data | limitations of the study added at page 7-line 21-27 |
|  | 6. Conclusion The conclusion appropriately summarizes the main findings |  |
|  | 7. References References appear appropriate. Please ensure formatting is consistent with PLOS ONE guidelines |  |

Yours Sincerely,

Richard Machoka

Corresponding author.
