# Supplementary material for: Co-occurrence of antibiotic residues and Antimicrobial resistance genes in animal manure and agricultural soils from Machakos, Kiambu, and Kajiado Counties, Kenya
Source: PLoS One. 2026 Jul 24;21(7):e0337311. doi: 10.1371/journal.pone.0337311 (PMC13399453; doi:10.1371/journal.pone.0337311)
Supplement: Attachment — Submitted filename: Reviewer Comments.docx [file pone.0337311.s004.docx]

**Reviewer Comments**

**Overall Assessment**

This manuscript addresses an important public health and environmental issue by examining antibiotic residues and/or ARG distribution across three counties in Kenya. The topic is both timely and relevant, particularly within the context of the One Health framework. The inclusion of multiple livestock systems and geographic comparison is a strength. However, several methodological and reporting issues require clarification and stronger acknowledgment before the manuscript is suitable for publication.

**Minor Comments**

**1. Limitations Section Requires Strengthening**

The current limitations section does not fully acknowledge key methodological constraints of the study.

- **Cross-sectional design:** The study is cross-sectional, with samples collected at a single time point. This design limits causal inference and does not allow assessment of temporal or seasonal variability. While future seasonal studies are recommended, the manuscript should explicitly state how the present design constrains interpretation.

**Recommendation**

The manuscript addresses an important topic but requires clarification of methodological limitations. I recommend revision before acceptance.
