# Supplementary material for: Co-occurrence of antibiotic residues and Antimicrobial resistance genes in animal manure and agricultural soils from Machakos, Kiambu, and Kajiado Counties, Kenya
Source: PLoS One. 2026 Jul 24;21(7):e0337311. doi: 10.1371/journal.pone.0337311 (PMC13399453; doi:10.1371/journal.pone.0337311)
Supplement: Attachment — Submitted filename: Response to Reviewers_3.docx [file pone.0337311.s005.docx]

|  | Editors Comments | Authors Response |
| --- | --- | --- |
| 1. | Please expand the discussion of the results presented on page 20 (lines 24–33) and page 21 (lines 1–25) by interpreting them in the context of recent similar published studies and highlighting the implications of your findings. The references should be within the last five years. | Addressed by adding a new paragraph in page 15 line 24-33 and page 16 line 1-7. The paragraphs give additional information of the effects of antibiotics residues to the environment from soil, plants and even other organisms. |
| 2 | Please clearly highlight the major limitations of the study. These limitations should be incorporated into the final paragraph of the **Discussion** section rather than presented as a separate subsection. | Addressed by moving the limitation and making it the last paragraph in the discussion section. Page 17 line 6-14. |
|  |  |  |

PONE-D-25-59999

Co-occurrence of Antibiotic Residues and Antimicrobial Resistance Genes in Animal Manure and Agricultural Soils from Machakos, Kiambu, and Kajiado Counties, Kenya
