# Supplementary material for: Co-occurrence of antibiotic residues and Antimicrobial resistance genes in animal manure and agricultural soils from Machakos, Kiambu, and Kajiado Counties, Kenya
Source: PLoS One. 2026 Jul 24;21(7):e0337311. doi: 10.1371/journal.pone.0337311 (PMC13399453; doi:10.1371/journal.pone.0337311)
Supplement: Peer review history

# Peer review: Decision Letter 0

**DOI:** 10.1371/journal.pone.0337311.r001

**Authors:** Mabel Kamweli Aworh

24 Nov 2025

PONE-D-25-59999

Co-occurrence of Antibiotic Residues and Antimicrobial Resistance Genes in Animal Manure and Agricultural Soils from Machakos, Kiambu, and Kajiado Counties, Kenya

PLOS ONE

Dear Dr. Machoka,

Thank you for submitting your manuscript to PLOS ONE. After careful consideration, we feel that it has merit but does not fully meet PLOS ONE’s publication criteria as it currently stands. Therefore, we invite you to submit a revised version of the manuscript that addresses the points raised during the review process.

Please submit your revised manuscript by Jan 08 2026 11:59PM.  If you will need more time than this to complete your revisions, please reply to this message or contact the journal office at plosone@plos.org. When you're ready to submit your revision, log on to https://www.editorialmanager.com/pone/ and select the 'Submissions Needing Revision' folder to locate your manuscript file.

Please include the following items when submitting your revised manuscript:

- A rebuttal letter that responds to each point raised by the academic editor and reviewer(s). You should upload this letter as a separate file labeled 'Response to Reviewers'.
- A marked-up copy of your manuscript that highlights changes made to the original version. You should upload this as a separate file labeled 'Revised Manuscript with Track Changes'.
- An unmarked version of your revised paper without tracked changes. You should upload this as a separate file labeled 'Manuscript'.

If you would like to make changes to your financial disclosure, please include your updated statement in your cover letter. Guidelines for resubmitting your figure files are available below the reviewer comments at the end of this letter.

If applicable, we recommend that you deposit your laboratory protocols in protocols.io to enhance the reproducibility of your results. Protocols.io assigns your protocol its own identifier (DOI) so that it can be cited independently in the future. For instructions see: https://journals.plos.org/plosone/s/submission-guidelines#loc-laboratory-protocols. Additionally, PLOS ONE offers an option for publishing peer-reviewed Lab Protocol articles, which describe protocols hosted on protocols.io. Read more information on sharing protocols at https://plos.org/protocols?utm_medium=editorial-email&utm_source=authorletters&utm_campaign=protocols.

We look forward to receiving your revised manuscript.

Kind regards,

Mabel Kamweli Aworh, DVM, MPH, PhD. FCVSN

Academic Editor

PLOS ONE

**Journal Requirements:**

When submitting your revision, we need you to address these additional requirements.

1. Please ensure that your manuscript meets PLOS ONE's style requirements, including those for file naming. The PLOS ONE style templates can be found at

https://journals.plos.org/plosone/s/file?id=wjVg/PLOSOne_formatting_sample_main_body.pdf and

https://journals.plos.org/plosone/s/file?id=ba62/PLOSOne_formatting_sample_title_authors_affiliations.pdf

2. Thank you for stating the following financial disclosure:

“This work was funded by the Alexander von Humbold Foundation under Reference number. 3.4 - 1071930 - KEN – IP”

Please state what role the funders took in the study.  If the funders had no role, please state: "The funders had no role in study design, data collection and analysis, decision to publish, or preparation of the manuscript."

If this statement is not correct you must amend it as needed.

Please include this amended Role of Funder statement in your cover letter; we will change the online submission form on your behalf.

3. Please note that funding information should not appear in any section or other areas of your manuscript. We will only publish funding information present in the Funding Statement section of the online submission form. Please remove any funding-related text from the manuscript.

4. PLOS requires an ORCID iD for the corresponding author in Editorial Manager on papers submitted after December 6th, 2016. Please ensure that you have an ORCID iD and that it is validated in Editorial Manager. To do this, go to ‘Update my Information’ (in the upper left-hand corner of the main menu), and click on the Fetch/Validate link next to the ORCID field. This will take you to the ORCID site and allow you to create a new iD or authenticate a pre-existing iD in Editorial Manager.

5. We note that Figure 1 in your submission contain map  images which may be copyrighted. All PLOS content is published under the Creative Commons Attribution License (CC BY 4.0), which means that the manuscript, images, and Supporting Information files will be freely available online, and any third party is permitted to access, download, copy, distribute, and use these materials in any way, even commercially, with proper attribution. For these reasons, we cannot publish previously copyrighted maps or satellite images created using proprietary data, such as Google software (Google Maps, Street View, and Earth). For more information, see our copyright guidelines: http://journals.plos.org/plosone/s/licenses-and-copyright.

We require you to either (1) present written permission from the copyright holder to publish these figures specifically under the CC BY 4.0 license, or (2) remove the figures from your submission:

1. You may seek permission from the original copyright holder of Figure(s) [#] to publish the content specifically under the CC BY 4.0 license.

We recommend that you contact the original copyright holder with the Content Permission Form (http://journals.plos.org/plosone/s/file?id=7c09/content-permission-form.pdf) and the following text:

“I request permission for the open-access journal PLOS ONE to publish XXX under the Creative Commons Attribution License (CCAL) CC BY 4.0 (http://creativecommons.org/licenses/by/4.0/). Please be aware that this license allows unrestricted use and distribution, even commercially, by third parties. Please reply and provide explicit written permission to publish XXX under a CC BY license and complete the attached form.”

Please upload the completed Content Permission Form or other proof of granted permissions as an "Other" file with your submission.

In the figure caption of the copyrighted figure, please include the following text: “Reprinted from [ref] under a CC BY license, with permission from [name of publisher], original copyright [original copyright year].”

2. If you are unable to obtain permission from the original copyright holder to publish these figures under the CC BY 4.0 license or if the copyright holder’s requirements are incompatible with the CC BY 4.0 license, please either i) remove the figure or ii) supply a replacement figure that complies with the CC BY 4.0 license. Please check copyright information on all replacement figures and update the figure caption with source information. If applicable, please specify in the figure caption text when a figure is similar but not identical to the original image and is therefore for illustrative purposes only.

The following resources for replacing copyrighted map figures may be helpful:

USGS National Map Viewer (public domain): http://viewer.nationalmap.gov/viewer/

The Gateway to Astronaut Photography of Earth (public domain): http://eol.jsc.nasa.gov/sseop/clickmap/

Maps at the CIA (public domain): https://www.cia.gov/library/publications/the-world-factbook/index.html and https://www.cia.gov/library/publications/cia-maps-publications/index.html

NASA Earth Observatory (public domain): http://earthobservatory.nasa.gov/

Landsat: http://landsat.visibleearth.nasa.gov/

USGS EROS (Earth Resources Observatory and Science (EROS) Center) (public domain): http://eros.usgs.gov/#

Natural Earth (public domain): http://www.naturalearthdata.com/

6. Please include captions for your Supporting Information files at the end of your manuscript, and update any in-text citations to match accordingly. Please see our Supporting Information guidelines for more information: http://journals.plos.org/plosone/s/supporting-information.

7. If the reviewer comments include a recommendation to cite specific previously published works, please review and evaluate these publications to determine whether they are relevant and should be cited. There is no requirement to cite these works unless the editor has indicated otherwise.

**Additional Editor Comments:**

In addition to addressing all the concerns of the reviewers please fix the following issues;

1. **Condense the Conclusion.**  The conclusion mixes results, interpretation, and recommendations. It should emphasize the **main takeaway**  and then briefly state **implications.** It contains too much technical detail (e.g., gene ratios) distracts from the broader message.  The conclusion should focus on 3–4 sentences:
2.
  - Broader significance (One Health approach).
  - Policy/practice recommendation (better regulation, manure treatment, surveillance).
  - Key implication (livestock systems as reservoirs of ARGs).
  - Main finding (spatial heterogeneity, link between antibiotic use and contamination).
3. **Move Details to the Discussion**
  - Specifics like *tetQ:16s rRNA ratios* , *gene names* , and *manure treatment methods*  should remain in the discussion, not the conclusion.
4. **Add Study Limitations**
  - Please highlight key limitations of the study in the last paragraph of the discussion, not the conclusion. Examples:
    Sampling limited to three counties.
  - Cross‑sectional design (no temporal trends).
  - Lack of direct measurement of antibiotic usage practices.
  - Possible underestimation/overestimation of ARG diversity due to methodology.
5. **Improve the language as there are too many gramatical and typographical errors**
  - Correct typos and streamline sentences for clarity.
  - Replace “there is need for…” with more formal phrasing like “We recommend…” or “Future work should…”

[Note: HTML markup is below. Please do not edit.]

Reviewers' comments:

Reviewer's Responses to Questions

**Comments to the Author**

1. Is the manuscript technically sound, and do the data support the conclusions?

The manuscript must describe a technically sound piece of scientific research with data that supports the conclusions. Experiments must have been conducted rigorously, with appropriate controls, replication, and sample sizes. The conclusions must be drawn appropriately based on the data presented.

Reviewer #1: Partly

Reviewer #2: Yes

Reviewer #3: Partly

Reviewer #4: Partly

**********

2. Has the statistical analysis been performed appropriately and rigorously?

Reviewer #1: Yes

Reviewer #2: Yes

Reviewer #3: No

Reviewer #4: No

**********

3. Have the authors made all data underlying the findings in their manuscript fully available?

The PLOS Data policy requires authors to make all data underlying the findings described in their manuscript fully available without restriction, with rare exception (please refer to the Data Availability Statement in the manuscript PDF file). The data should be provided as part of the manuscript or its supporting information, or deposited to a public repository. For example, in addition to summary statistics, the data points behind means, medians and variance measures should be available. If there are restrictions on publicly sharing data—e.g. participant privacy or use of data from a third party—those must be specified.

Reviewer #1: Yes

Reviewer #2: Yes

Reviewer #3: No

Reviewer #4: Yes

**********

4. Is the manuscript presented in an intelligible fashion and written in standard English?

PLOS ONE does not copyedit accepted manuscripts, so the language in submitted articles must be clear, correct, and unambiguous. Any typographical or grammatical errors should be corrected at revision, so please note any specific errors here.

Reviewer #1: Yes

Reviewer #2: Yes

Reviewer #3: No

Reviewer #4: Yes

**********

5. Review Comments to the Author

Please use the space provided to explain your answers to the questions above. You may also include additional comments for the author, including concerns about dual publication, research ethics, or publication ethics. (Please upload your review as an attachment if it exceeds 20,000 characters)

Reviewer #1: In page 2, lines 25 - 27 the author does not specify the geographical region that the high concentrations were found, and the reference (Kumar et al 2005) is old. Lines 25 - 27 of page 2 forms the crux of the study aims, so a more robust literature review is required.

In page 2 lines 23 and 24, it is not clear which category sulphonamides and acetic conjugates fall into. I recommend rewording the sentence to be clearer. It can be written as "some metabolites such as... end up being more potent..." or "some metabolites end up being more potent... while others such as..."

Data analysis - The alpha value was not defined in the study.

In page 12, line 2 the wrong p value was entered and should be corrected to 0.0547.

The reference appears to be missing in page 21 lines 15,16 and 17.

In page 21, line 6 "and" is missing in the sentence "resistance) were not detected [and] rarely detected, respectively, indicating either limited exposure"

General comments - It would be beneficial for readers to have more information on the specific antibiotic compounds in the background.

Reviewer #2: General Assessment

This manuscript presents a topic highly relevant to global AMR surveillance efforts and aligns with One Health objectives. Overall, the study demonstrates scientific soundness, describes the methodology, and makes a meaningful contribution to the literature on environmental AMR dissemination in sub-Saharan Africa.

Strengths of the Manuscript

1. Novelty and Significance

The study provides uncommon, region-specific data on the combined assessment of antibiotic residues and ARGs in both manure and agricultural soil, an approach that is still limited across African agricultural systems. Such dual assessment allows strong inferences about selective pressure and environmental resistome dynamics.

2. Methodological Rigor

The sampling design is generally appropriate and justified.

The extraction and LC-MS/MS procedures are well described and follow standardized protocols.

qPCR quantification and normalization to 16S rRNA strengthen the interpretation of resistome profiles.

Ethical approval is stated.

However,

a. How was the soil sample collected?

b. What type of container was used to collect the soil sample: sterile or nonsterile?

c. At what depth of soil was it collected? Was it topsoil or subsoil?

There should be a subtitle for sample collection.

3. Analysis and Interpretation

The study integrates residue quantification, absolute and relative ARG abundance, multivariate analysis (PCoA), and correlation modeling. The triangulation between residue concentration and gene abundance is a major advantage that strengthens the conclusions.

Areas for Improvement

1. Statistical Analysis

The three-way ANOVA was appropriately applied; however:

Some marginally significant values (p = 0.06–0.10) are interpreted as meaningful. These should be described more cautiously.

Provide effect sizes to better understand the magnitude of differences.

Clarify whether ANOVA assumptions (normality, homogeneity) were checked.

2. Data Availability

The manuscript does not provide full raw datasets (individual sample-level concentrations, Ct values, and standard curves).

Recommendation: Upload processed and raw datasets to a public repository (Figshare, OSF, Dryad) to comply with journal policy.

3. Clarity and Organization.

The manuscript is generally well written but includes several typographical and grammatical issues.

Examples:

“the sample was be calculated” is used instead of “the sample size was calculated” (materials and methods)

The word "inn" is incorrectly used instead of "in" in the conclusion.

"Wo" is used instead of "who" in the acknowledgement section.

These represent only a few examples. The manuscript contains many more areas where grammar, phrasing, and overall readability need improvement.

Some long paragraphs can be broken into shorter ones to improve readability.

4. Figures and Tables.

All figures should include units and error bars where applicable.

Supplementary materials should be referenced more consistently.

5. Methodological Details

For reproducibility:

Provide catalog numbers for reagents and LC-MS/MS standards used.

Include limit of detection (LOD) and limit of quantification (LOQ) for each antibiotic.

Clarify whether manure samples were fresh, composted, or mixed; this influences residue degradation kinetics.

1. How was the soil sample collected? 2. What type of container was used to collect the soil sample, sterile or non-sterile? 3. At what depth of soil was it collected? Was it topsoil or subsoil?

There should be a subtitle for sample collection.

6. Interpretation of Results

The conclusions are supported by the data; however, there are some limitations to consider:

The manuscript occasionally overgeneralizes findings; for example, attributing all high ARG abundance exclusively to antibiotic pressure without considering co-selection by metals or other stressors.

Provide possible explanations for county-specific variations (differences in farming intensity, soil properties, waste handling, etc.).

7. Literature Context

The manuscript cites relevant global and regional literature but could benefit from:

The manuscript could benefit from more discussion on environmental drivers of ARG persistence, such as organic matter, soil pH, and manure management practices.

The manuscript also needs to incorporate recent studies on soil and manure resistance conducted in Africa between 2019 and 2025.

Finally, I recommend Major Revision (Encouraged to Resubmit).

The study is strong and worthy of publication but requires improvements in data transparency, polishing of language, and clearer presentation of statistical results. Addressing these issues will enable the manuscript to significantly contribute to AMR research and environmental health in sub-Saharan Africa.

Reviewer #3: Summary and overall impression

The manuscript titled "Co-occurrence of Antibiotic Residues and Antimicrobial Resistance Genes in Animal Manure and Agricultural Soils from Machakos, Kiambu, and Kajiado Counties, Kenya" is very relevant and useful; however, it lacks statistical rigor and needs a proper description of the methodology as well as an ideal interpretation of the results.

Major Issues

1. Multiple sampling methods were not appropriate. The purposive sampling and convenience sampling are non-random, which contradicts the stratified random sampling which introduces systemic bias. The manuscript does not have a section for limitiation to acknowledge this.

2. The sample size calculation was inappropriate for this type of study; the study was exploratory and not inferential. Pooling of the samples into 18 samples alienates on-site differences, and devalues the following three-way ANOVA. The county-level comparisons are not valid because the sampling was pooled, unreplicated, and purposively biased. Also, the statistical software used to calculate the averages, make bar charts, and even perform principal component analysis and other diagrames was not mentioned, nor was the version stated.

Minor

The abstract states, “A total of 180 samples from 30 farms across the three counties were collected and pooled into 18 samples", The specific type of sample was not stated. There were also grammatical errors, few references for the discussion, and no objective in the introduction, even though it was stated in the abstract. Diagrams are not well labelled to match the in-text writings. No limitation stated and novelty of this paper is unknown. The title and results mention antimicrobial-resistant genes (ARGs) and antibiotic residues, but the extensive literature on the ARGs was not described, which makes the literature review incomplete.

Recommendations: Major Revision

Given the issues highlighted above, particularly the sampling inconsistencies, invalid ANOVA, incomplete methodological detail, and absence of an ARG-focused literature review, the manuscript requires substantial revision. The authors should address all concerns outlined above and reconsider the interpretation of their findings within the limitations of an exploratory design.

Reviewer #4: Review Comments to the Author

Recommendation: Major Revision

This manuscript examines the co-occurrence of antibiotic residues and antimicrobial resistance genes (ARGs) in livestock manure and agricultural soils across three Kenyan counties. The topic is highly relevant to One Health research, and the study provides valuable data from an understudied setting. However, several areas need clarification or strengthening before the work is ready for publication.

1. Introduction

The introduction is well structured and provides helpful context. To improve clarity, the authors should explicitly state the main objective of the study in the final paragraph.

2. Methods

The methods are generally detailed, but several issues require attention:

• Sample pooling: The manuscript reports that 180 samples were collected but later pooled into 18 composite samples. The rationale for this approach is not clearly explained. Pooling can substantially reduce variability and may affect the validity of the statistical analysis.

• ANOVA assumptions: Three-way ANOVA assumes independent observations. Because the data were pooled, this assumption may not hold. The authors should justify the approach and discuss its limitations.

3. Results

The results are clearly described and supported by visualizations. I recommend:

• Reporting p-values consistently (e.g., to three decimal places) in Table 1.

• Ensuring all figures are high-resolution and labeled clearly.

4. Discussion

The discussion is informative and connects findings to existing literature. However:

• Several paragraphs repeat results rather than interpreting them.

• The authors could more fully explain potential reasons for county-level differences, such as farming intensity or antibiotic access.

5. Limitations

Important limitations are not fully acknowledged. The authors should consider adding:

• The impact of sample pooling on statistical power and interpretation.

• The cross-sectional design and lack of temporal or seasonal data.

6. Conclusion

The conclusion appropriately summarizes the main findings

7. References

References appear appropriate. Please ensure formatting is consistent with PLOS ONE guidelines.

Overall Assessment

This manuscript addresses an important public health and environmental issue and provides useful data. However, clarifying the rationale for sample pooling, strengthening the statistical interpretation, improving figure presentation, and expanding the limitations section are necessary.

I recommend a Major Revision.

**********

6. PLOS authors have the option to publish the peer review history of their article (what does this mean?). If published, this will include your full peer review and any attached files.

If you choose “no”, your identity will remain anonymous but your review may still be made public.

**Do you want your identity to be public for this peer review?**  For information about this choice, including consent withdrawal, please see our Privacy Policy.

Reviewer #1: **Yes:**  Uzoma Emmanuella Adebayo

Reviewer #2: No

Reviewer #3: No

Reviewer #4: No

**********

[NOTE: If reviewer comments were submitted as an attachment file, they will be attached to this email and accessible via the submission site. Please log into your account, locate the manuscript record, and check for the action link "View Attachments". If this link does not appear, there are no attachment files.]

To ensure your figures meet our technical requirements, please review our figure guidelines: https://journals.plos.org/plosone/s/figures

You may also use PLOS’s free figure tool, NAAS, to help you prepare publication quality figures: https://journals.plos.org/plosone/s/figures#loc-tools-for-figure-preparation.

NAAS will assess whether your figures meet our technical requirements by comparing each figure against our figure specifications.

<qb-div data-qb-element="re-enable-flow" style="z-index: 2147483647; max-width: 1px; max-height: 1px; box-sizing: border-box; position: fixed; top: 10px; right: 10px;">--><qb-div style="all: initial !important;"></qb-div>--></qb-div>

# Author comment: Author response to Decision Letter 1

**DOI:** 10.1371/journal.pone.0337311.r002

16 Feb 2026

Funders role

This work was funded by the Alexander von Humbold Foundation under Reference number. 3.4 - 1071930 - KEN – IP. The funders had no role in study design, data collection and analysis, decision to publish, or preparation of the manuscript.

Figure 1: Map

Base maps, boundaries and shape files of Kenyan map and administrative boundaries of the Counties were derived from GADM data version 4.1 (https://gadm.org) and the maps were generated using ArcGIS Version 10.2.2 (http://desktop.arcgis.com/en/arcmap) advanced license) courtesy of Samuel Owaka.

Attachment Submitted filename: Response to reviewers.docx

# Peer review: Decision Letter 1

**DOI:** 10.1371/journal.pone.0337311.r003

**Authors:** Mabel Kamweli Aworh

12 Mar 2026

PONE-D-25-59999R1

Co-occurrence of Antibiotic Residues and Antimicrobial Resistance Genes in Animal Manure and Agricultural Soils from Machakos, Kiambu, and Kajiado Counties, Kenya

PLOS One

Dear Dr. Machoka,

Thank you for submitting your manuscript to PLOS ONE. After careful consideration, we feel that it has merit but does not fully meet PLOS ONE’s publication criteria as it currently stands. Therefore, we invite you to submit a revised version of the manuscript that addresses the points raised during the review process.

Please submit your revised manuscript by Apr 26 2026 11:59PM. If you will need more time than this to complete your revisions, please reply to this message or contact the journal office at plosone@plos.org. When you're ready to submit your revision, log on to https://www.editorialmanager.com/pone/ and select the 'Submissions Needing Revision' folder to locate your manuscript file.

Please include the following items when submitting your revised manuscript:

- A letter that responds to each point raised by the academic editor and reviewer(s). You should upload this letter as a separate file labeled 'Response to Reviewers'.
- A marked-up copy of your manuscript that highlights changes made to the original version. You should upload this as a separate file labeled 'Revised Manuscript with Track Changes'.
- An unmarked version of your revised paper without tracked changes. You should upload this as a separate file labeled 'Manuscript'.

-->If you would like to make changes to your financial disclosure, please include your updated statement in your cover letter. Guidelines for resubmitting your figure files are available below the reviewer comments at the end of this letter.

If applicable, we recommend that you deposit your laboratory protocols in protocols.io to enhance the reproducibility of your results. Protocols.io assigns your protocol its own identifier (DOI) so that it can be cited independently in the future. For instructions see: https://journals.plos.org/plosone/s/submission-guidelines#loc-laboratory-protocols. Additionally, PLOS ONE offers an option for publishing peer-reviewed Lab Protocol articles, which describe protocols hosted on protocols.io. Read more information on sharing protocols at https://plos.org/protocols?utm_medium=editorial-email&utm_source=authorletters&utm_campaign=protocols.

We look forward to receiving your revised manuscript.

Kind regards,

Mabel Kamweli Aworh, DVM, MPH, PhD. FCVSN

Academic Editor

PLOS One

Journal Requirements:

If the reviewer comments include a recommendation to cite specific previously published works, please review and evaluate these publications to determine whether they are relevant and should be cited. There is no requirement to cite these works unless the editor has indicated otherwise.

Please review your reference list to ensure that it is complete and correct. If you have cited papers that have been retracted, please include the rationale for doing so in the manuscript text, or remove these references and replace them with relevant current references. Any changes to the reference list should be mentioned in the rebuttal letter that accompanies your revised manuscript. If you need to cite a retracted article, indicate the article’s retracted status in the References list and also include a citation and full reference for the retraction notice.

Additional Editor Comments:

In addition to addressing the comments from the reviewers, please attend to the following minor issues:

1. Please expand the discussion of the results presented on page 20 (lines 24–33) and page 21 (lines 1–25) by interpreting them in the context of recent similar published studies and highlighting the implications of your findings. The references should be within the last five years.

2. Please clearly highlight the major limitations of the study. These limitations should be incorporated into the final paragraph of the **Discussion**  section rather than presented as a separate subsection.

[Note: HTML markup is below. Please do not edit.]

Reviewers' comments:

Reviewer's Responses to Questions

**Comments to the Author**

1. If the authors have adequately addressed your comments raised in a previous round of review and you feel that this manuscript is now acceptable for publication, you may indicate that here to bypass the “Comments to the Author” section, enter your conflict of interest statement in the “Confidential to Editor” section, and submit your "Accept" recommendation.

Reviewer #2: All comments have been addressed

Reviewer #3: All comments have been addressed

Reviewer #4: (No Response)

**********

2. Is the manuscript technically sound, and do the data support the conclusions?

The manuscript must describe a technically sound piece of scientific research with data that supports the conclusions. Experiments must have been conducted rigorously, with appropriate controls, replication, and sample sizes. The conclusions must be drawn appropriately based on the data presented.

Reviewer #2: Yes

Reviewer #3: Yes

Reviewer #4: Yes

**********

3. Has the statistical analysis been performed appropriately and rigorously?

Reviewer #2: Yes

Reviewer #3: Yes

Reviewer #4: Yes

**********

4. Have the authors made all data underlying the findings in their manuscript fully available?

The PLOS Data policy requires authors to make all data underlying the findings described in their manuscript fully available without restriction, with rare exception (please refer to the Data Availability Statement in the manuscript PDF file). The data should be provided as part of the manuscript or its supporting information, or deposited to a public repository. For example, in addition to summary statistics, the data points behind means, medians and variance measures should be available. If there are restrictions on publicly sharing data—e.g. participant privacy or use of data from a third party—those must be specified.

Reviewer #2: Yes

Reviewer #3: No

Reviewer #4: Yes

**********

5. Is the manuscript presented in an intelligible fashion and written in standard English?

PLOS ONE does not copyedit accepted manuscripts, so the language in submitted articles must be clear, correct, and unambiguous. Any typographical or grammatical errors should be corrected at revision, so please note any specific errors here.

Reviewer #2: Yes

Reviewer #3: Yes

Reviewer #4: Yes

**********

6. Review Comments to the Author

Please use the space provided to explain your answers to the questions above. You may also include additional comments for the author, including concerns about dual publication, research ethics, or publication ethics. (Please upload your review as an attachment if it exceeds 20,000 characters)

Reviewer #2: I appreciate the authors' efforts in revising the work and responding to reviewer comments. The revisions improved the manuscript, and the issues identified in my prior review were effectively addressed. The addition of a subsection describing sample collection explains the procedures for collecting soil and manure samples, increases method transparency, and facilitates replication. The authors also explained the statistical analysis and validated that the ANOVA assumptions were met, and the raw dataset was uploaded to the Dryad repository. In addition, typographical errors have been fixed, figures and tables have been updated, and the discussion has been expanded to account for environmental issues such as soil characteristics and differences in agricultural practices across counties. I have no major concerns and believe the manuscript is appropriate for publication.

Reviewer #3: Summary of the Study

The manuscript, titled "Co-occurrence of Antibiotic Residues and Antimicrobial Resistance Genes in Animal Manure and Agricultural Soils from Machakos, Kiambu, and Kajiado Counties, Kenya," addresses a significant global health issue concerning environmental antibiotic contamination and the potential dissemination of antimicrobial resistance through agricultural systems. This topic is highly relevant to environmental microbiology, agricultural sustainability, and public health. The revised version has addressed several concerns from the previous review, including clarifying sampling procedures, correcting typographical errors, defining statistical significance thresholds, and including additional methodological details.

Strengths

The study advances understanding of environmental reservoirs of antimicrobial resistance within agricultural systems, which is an emerging global health concern. Given the limited data on environmental antibiotic contamination in East Africa, this research provides valuable baseline information for Kenya. The methodological clarification has been improved, and the application of LC-ESI-triple quadrupole mass spectrometry for antibiotic residue quantification is appropriate and aligns with standard analytical practices.

Major Comments

The manuscript states that 180 individual samples were collected and subsequently pooled into 18 composite samples prior to analysis. However, it does not adequately describe the number of individual samples contributing to each composite, whether equal mass or volume from each sample was used, or the rationale for pooling prior to analysis.

The manuscript reports several marginal p-values (approximately 0.06 to 0.10) from the three-way ANOVA analysis and interprets these as meaningful trends. The text does not clearly differentiate between statistically significant results (p < 0.05) and non-significant trends.

Although the authors state that the raw datasets have been uploaded to the Dryad repository, these datasets are not yet publicly accessible due to repository fees. The dataset should be made publicly accessible prior to publication and accompanied by a DOI or accession number. Alternatively, the relevant datasets could be provided as supplementary files.

Minor Comments

Several sentences in the introduction and discussion remain somewhat lengthy and would benefit from stylistic refinement to enhance readability. The manuscript would also benefit from a brief explanation of how antibiotic usage patterns among farms may influence the observed residue levels. Additionally, while the limitations section appropriately addresses the effects of sample pooling, this discussion could be expanded to further emphasize how pooling may affect the sensitivity of detection of antimicrobial resistance genes.

Recommendation

Minor Revision: The manuscript addresses an important topic and provides valuable data on environmental antibiotic contamination in agriculture. The authors should clarify and address the issues identified. Most reviewer concerns have been addressed in the revised version; however, remaining issues, particularly regarding the sample pooling strategy and data availability, should be resolved prior to publication.

Reviewer #4: (No Response)

**********

7. PLOS authors have the option to publish the peer review history of their article (what does this mean?). If published, this will include your full peer review and any attached files.

If you choose “no”, your identity will remain anonymous but your review may still be made public.

**Do you want your identity to be public for this peer review?**  For information about this choice, including consent withdrawal, please see our Privacy Policy.

Reviewer #2: No

Reviewer #3: No

Reviewer #4: No

**********

[NOTE: If reviewer comments were submitted as an attachment file, they will be attached to this email and accessible via the submission site. Please log into your account, locate the manuscript record, and check for the action link "View Attachments". If this link does not appear, there are no attachment files.]

To ensure your figures meet our technical requirements, please review our figure guidelines: https://journals.plos.org/plosone/s/figures

You may also use PLOS’s free figure tool, NAAS, to help you prepare publication quality figures: https://journals.plos.org/plosone/s/figures#loc-tools-for-figure-preparation.

NAAS will assess whether your figures meet our technical requirements by comparing each figure against our figure specifications.

Attachment Submitted filename: Reviewer Comments.docx

# Author comment: Author response to Decision Letter 2

**DOI:** 10.1371/journal.pone.0337311.r004

16 Mar 2026

Most of the reviewers' comments have been addressed.

Attachment Submitted filename: Response to Reviewers_3.docx

# Peer review: Decision Letter 2

**DOI:** 10.1371/journal.pone.0337311.r005

**Authors:** Mabel Kamweli Aworh

15 Apr 2026

PONE-D-25-59999R2

Co-occurrence of Antibiotic Residues and Antimicrobial Resistance Genes in Animal Manure and Agricultural Soils from Machakos, Kiambu, and Kajiado Counties, Kenya

PLOS One

Dear Dr. Machoka,

Thank you for submitting your manuscript to PLOS ONE. After careful consideration, we feel that it has merit but does not fully meet PLOS ONE’s publication criteria as it currently stands. Therefore, we invite you to submit a revised version of the manuscript that addresses the points raised during the review process.

Please submit your revised manuscript by May 30 2026 11:59PM. If you will need more time than this to complete your revisions, please reply to this message or contact the journal office at plosone@plos.org. When you're ready to submit your revision, log on to https://www.editorialmanager.com/pone/ and select the 'Submissions Needing Revision' folder to locate your manuscript file.

Please include the following items when submitting your revised manuscript:

- A letter that responds to each point raised by the academic editor and reviewer(s). You should upload this letter as a separate file labeled 'Response to Reviewers'.
- A marked-up copy of your manuscript that highlights changes made to the original version. You should upload this as a separate file labeled 'Revised Manuscript with Track Changes'.
- An unmarked version of your revised paper without tracked changes. You should upload this as a separate file labeled 'Manuscript'.

If you would like to make changes to your financial disclosure, please include your updated statement in your cover letter. Guidelines for resubmitting your figure files are available below the reviewer comments at the end of this letter.

If applicable, we recommend that you deposit your laboratory protocols in protocols.io to enhance the reproducibility of your results. Protocols.io assigns your protocol its own identifier (DOI) so that it can be cited independently in the future. For instructions see: https://journals.plos.org/plosone/s/submission-guidelines#loc-laboratory-protocols. Additionally, PLOS ONE offers an option for publishing peer-reviewed Lab Protocol articles, which describe protocols hosted on protocols.io. Read more information on sharing protocols at https://plos.org/protocols?utm_medium=editorial-email&utm_source=authorletters&utm_campaign=protocols.

As the corresponding author, your ORCID iD is verified in the submission system and will appear in the published article. PLOS supports the use of ORCID, and we encourage all coauthors to register for an ORCID iD and use it as well. Please encourage your coauthors to verify their ORCID iD within the submission system before final acceptance, as unverified ORCID iDs will not appear in the published article. *Only*  the individual author can complete the verification step; PLOS staff *cannot*  verify ORCID iDs on behalf of authors.

We look forward to receiving your revised manuscript.

Kind regards,

Mabel Kamweli Aworh, DVM, MPH, PhD. FCVSN

Academic Editor

PLOS One

Journal Requirements:

If the reviewer comments include a recommendation to cite specific previously published works, please review and evaluate these publications to determine whether they are relevant and should be cited. There is no requirement to cite these works unless the editor has indicated otherwise.

[Note: HTML markup is below. Please do not edit.]

Reviewers' comments:

Reviewer's Responses to Questions

**Comments to the Author**

1. If the authors have adequately addressed your comments raised in a previous round of review and you feel that this manuscript is now acceptable for publication, you may indicate that here to bypass the “Comments to the Author” section, enter your conflict of interest statement in the “Confidential to Editor” section, and submit your "Accept" recommendation.

Reviewer #3: (No Response)

Reviewer #4: All comments have been addressed

**********

2. Is the manuscript technically sound, and do the data support the conclusions?

The manuscript must describe a technically sound piece of scientific research with data that supports the conclusions. Experiments must have been conducted rigorously, with appropriate controls, replication, and sample sizes. The conclusions must be drawn appropriately based on the data presented.

Reviewer #3: Partly

Reviewer #4: Yes

**********

3. Has the statistical analysis been performed appropriately and rigorously?

Reviewer #3: No

Reviewer #4: Yes

**********

4. Have the authors made all data underlying the findings in their manuscript fully available?

The PLOS Data policy requires authors to make all data underlying the findings described in their manuscript fully available without restriction, with rare exception (please refer to the Data Availability Statement in the manuscript PDF file). The data should be provided as part of the manuscript or its supporting information, or deposited to a public repository. For example, in addition to summary statistics, the data points behind means, medians and variance measures should be available. If there are restrictions on publicly sharing data—e.g. participant privacy or use of data from a third party—those must be specified.

Reviewer #3: Yes

Reviewer #4: Yes

**********

5. Is the manuscript presented in an intelligible fashion and written in standard English?

PLOS ONE does not copyedit accepted manuscripts, so the language in submitted articles must be clear, correct, and unambiguous. Any typographical or grammatical errors should be corrected at revision, so please note any specific errors here.

Reviewer #3: No

Reviewer #4: Yes

**********

6. Review Comments to the Author

Please use the space provided to explain your answers to the questions above. You may also include additional comments for the author, including concerns about dual publication, research ethics, or publication ethics. (Please upload your review as an attachment if it exceeds 20,000 characters)

Reviewer #3: SUMMARY

This manuscript, titled “Co-occurrence of Antibiotic Residues and Antimicrobial Resistance Genes in Animal Manure and Agricultural Soils from Machakos, Kiambu, and Kajiado Counties, Kenya,” examines the spatial co-occurrence and distribution of antibiotic residues and antimicrobial resistance genes (ARGs) in livestock manure and agricultural soils from three Kenyan counties. The paper quantifies antibiotic residues and ARGs using LC-MS/MS and qPCR, and apply multivariate statistical analyses (PCoA, PCA, Spearman correlation) to assess relationships and patterns.

The study addresses a critical One Health issue, especially in low- and middle-income settings with limited antibiotic regulation. The identification of dominant tetracyclines and high levels of ARGs, such as aadA, ermB, and sul1, underscores urgent environmental and public health risks and underscores the need for immediate regulatory and mitigation strategies.

MAJOR COMMENTS

• The study design has significant limitations in terms of representativeness and analytical robustness. Convenience sampling, based on the discretion of farm owners, introduces potential selection bias and limits generalizability. Pooling 180 individual samples into 18 composites further reduces data resolution, obscures variability, and weakens the validity of subsequent statistical analyses.

• The statistical approach needs clarification and more cautious interpretation. Several results labeled as “marginally significant” (p-values between 0.05 and 0.10) are overinterpreted. There is no evidence that ANOVA assumptions were assessed or that multiple-comparison adjustments were made. Given the reduced effective sample size from pooling, the use of parametric analyses should be more thoroughly justified.

• Some conclusions exceed the study design's support. Attributing ARG distribution to “clear antibiotic use” and “environmental persistence” is not justified, as there is no direct antibiotic-use data or assessment of temporal dynamics. The findings indicate associations, not causality.

• Interpretation of results often relies on presumed antibiotic use patterns, but no primary data on antibiotic usage were collected. This limits the ability to contextualize findings, leading to speculative explanations.

• Reporting of antibiotic concentrations is inconsistent, with non-standard units for environmental samples and variation across sections.

MINOR COMMENT

• The manuscript would benefit from comprehensive language editing to correct grammatical inconsistencies, repetition, and unclear sentence structure.

• Methodological details, especially for qPCR and LC-MS/MS analyses, should be expanded to include validation parameters such as efficiency, recovery, and detection limits.

• Figures need clearer labeling and improved resolution, as some axes and legends currently reduce interpretability.

• The discussion section should be more structured to enhance readability and thematic coherence.

• Referencing should be standardized and updated to ensure consistency and alignment with journal formatting requirements.

RECOMMENDATION

Major Revision

The paper should clearly justify the sampling and pooling strategy, including details on pooling procedures, and explicitly acknowledge its impact on variability, representativeness, and inference. Avoid overinterpreting non-significant results, report statistical assumptions, clarify effective sample size, and refrain from attributing findings directly to antibiotic use without supporting data. Standardize units and ensure consistency throughout the manuscript. Clearly define the study’s unique contribution and its relevance to the current evidence base, and articulate its novelty relative to the existing literature.

Reviewer #4: The authors have satisfactorily addressed the concerns raised in the previous round of review, and the manuscript has improved significantly in clarity, rigor, and overall presentation. The study is technically sound, and the methods are appropriate to the research question. The data presented adequately support the conclusions, and the analyses are conducted with sufficient rigor.

The statistical approach is appropriate and clearly described, with results that are consistent and well interpreted. Any prior ambiguities have been resolved, and the linkage between the findings and conclusions is now clear. Additionally, the data availability statement meets the journal’s requirements, ensuring transparency and reproducibility.

The manuscript is well written, organized, and presented in clear, standard English, with only minor issues that do not affect comprehension.

**********

7. PLOS authors have the option to publish the peer review history of their article (what does this mean?). If published, this will include your full peer review and any attached files.

If you choose “no”, your identity will remain anonymous but your review may still be made public.

**Do you want your identity to be public for this peer review?**  For information about this choice, including consent withdrawal, please see our Privacy Policy.

Reviewer #3: No

Reviewer #4: No

**********

[NOTE: If reviewer comments were submitted as an attachment file, they will be attached to this email and accessible via the submission site. Please log into your account, locate the manuscript record, and check for the action link "View Attachments". If this link does not appear, there are no attachment files.]

To ensure your figures meet our technical requirements, please review our figure guidelines: https://journals.plos.org/plosone/s/figures

You may also use PLOS’s free figure tool, NAAS, to help you prepare publication quality figures: https://journals.plos.org/plosone/s/figures#loc-tools-for-figure-preparation.

NAAS will assess whether your figures meet our technical requirements by comparing each figure against our figure specifications.

# Author comment: Author response to Decision Letter 3

**DOI:** 10.1371/journal.pone.0337311.r006

14 May 2026

All the comments have been addressed.

Attachment Submitted filename: Response_to_reviewers_auresp_3.docx

# Peer review: Decision Letter 3

**DOI:** 10.1371/journal.pone.0337311.r007

**Authors:** Mabel Kamweli Aworh

10 Jun 2026

Co-occurrence of Antibiotic Residues and Antimicrobial Resistance Genes in Animal Manure and Agricultural Soils from Machakos, Kiambu, and Kajiado Counties, Kenya

PONE-D-25-59999R3

Dear Dr. Machoka,

We’re pleased to inform you that your manuscript has been judged scientifically suitable for publication and will be formally accepted for publication once it meets all outstanding technical requirements.

Within one week, you’ll receive an e-mail detailing the required amendments. When these have been addressed, you’ll receive a formal acceptance letter and your manuscript will be scheduled for publication.

An invoice will be generated when your article is formally accepted. Please note, if your institution has a publishing partnership with PLOS and your article meets the relevant criteria, all or part of your publication costs will be covered. Please make sure your user information is up-to-date by logging into Editorial Manager at Editorial Manager® and clicking the ‘Update My Information' link at the top of the page. For questions related to billing, please contact billing support.

If your institution or institutions have a press office, please notify them about your upcoming paper to help maximize its impact. If they’ll be preparing press materials, please inform our press team as soon as possible -- no later than 48 hours after receiving the formal acceptance. Your manuscript will remain under strict press embargo until 2 pm Eastern Time on the date of publication. For more information, please contact onepress@plos.org.

Kind regards,

Mabel Kamweli Aworh, DVM, MPH, PhD. FCVSN

Academic Editor

PLOS One

Additional Editor Comments (optional):

Reviewers' comments:

Reviewer's Responses to Questions

**Comments to the Author**

1. If the authors have adequately addressed your comments raised in a previous round of review and you feel that this manuscript is now acceptable for publication, you may indicate that here to bypass the “Comments to the Author” section, enter your conflict of interest statement in the “Confidential to Editor” section, and submit your "Accept" recommendation.

Reviewer #3: All comments have been addressed

Reviewer #4: All comments have been addressed

**********

2. Is the manuscript technically sound, and do the data support the conclusions?

The manuscript must describe a technically sound piece of scientific research with data that supports the conclusions. Experiments must have been conducted rigorously, with appropriate controls, replication, and sample sizes. The conclusions must be drawn appropriately based on the data presented.

Reviewer #3: Yes

Reviewer #4: Yes

**********

3. Has the statistical analysis been performed appropriately and rigorously?

Reviewer #3: Yes

Reviewer #4: Yes

**********

4. Have the authors made all data underlying the findings in their manuscript fully available?

The PLOS Data policy requires authors to make all data underlying the findings described in their manuscript fully available without restriction, with rare exception (please refer to the Data Availability Statement in the manuscript PDF file). The data should be provided as part of the manuscript or its supporting information, or deposited to a public repository. For example, in addition to summary statistics, the data points behind means, medians and variance measures should be available. If there are restrictions on publicly sharing data—e.g. participant privacy or use of data from a third party—those must be specified.

Reviewer #3: Yes

Reviewer #4: Yes

**********

5. Is the manuscript presented in an intelligible fashion and written in standard English?

PLOS ONE does not copyedit accepted manuscripts, so the language in submitted articles must be clear, correct, and unambiguous. Any typographical or grammatical errors should be corrected at revision, so please note any specific errors here.

Reviewer #3: Yes

Reviewer #4: Yes

**********

6. Review Comments to the Author

Please use the space provided to explain your answers to the questions above. You may also include additional comments for the author, including concerns about dual publication, research ethics, or publication ethics. (Please upload your review as an attachment if it exceeds 20,000 characters)

Reviewer #3: (No Response)

Reviewer #4: The revised manuscript satisfactorily addresses the reviewer comments from previous rounds. The study makes a valuable contribution to the understanding of antibiotic resistance in Kenyan agroecosystems and is now suitable for publication.

**********

7. PLOS authors have the option to publish the peer review history of their article (what does this mean?). If published, this will include your full peer review and any attached files.

If you choose “no”, your identity will remain anonymous but your review may still be made public.

**Do you want your identity to be public for this peer review?**  For information about this choice, including consent withdrawal, please see our Privacy Policy.

Reviewer #3: No

Reviewer #4: No

**********

# Editor report: Acceptance letter

**DOI:** 10.1371/journal.pone.0337311.r008

**Authors:** Mabel Kamweli Aworh

PONE-D-25-59999R3

PLOS One

Dear Dr. Machoka,

I'm pleased to inform you that your manuscript has been deemed suitable for publication in PLOS One. Congratulations! Your manuscript is now being handed over to our production team.

At this stage, our production department will prepare your paper for publication. This includes ensuring the following:

* All references, tables, and figures are properly cited

* All relevant supporting information is included in the manuscript submission,

* There are no issues that prevent the paper from being properly typeset

You will receive further instructions from the production team, including instructions on how to review your proof when it is ready. Please keep in mind that we are working through a large volume of accepted articles, so please give us a few days to review your paper and let you know the next and final steps.

Lastly, if your institution or institutions have a press office, please let them know about your upcoming paper now to help maximize its impact. If they'll be preparing press materials, please inform our press team within the next 48 hours. Your manuscript will remain under strict press embargo until 2 pm Eastern Time on the date of publication. For more information, please contact onepress@plos.org.

You will receive an invoice from PLOS for your publication fee after your manuscript has reached the completed accept phase. If you receive an email requesting payment before acceptance or for any other service, this may be a phishing scheme. Learn how to identify phishing emails and protect your accounts at https://explore.plos.org/phishing.

If we can help with anything else, please email us at customercare@plos.org.

Thank you for submitting your work to PLOS ONE and supporting open access.

Kind regards,

PLOS ONE Editorial Office Staff

on behalf of

Dr. Mabel Kamweli Aworh

Academic Editor

PLOS One
